# A recently transferred cluster of bacterial genes in *Trichomonas vaginalis -* lateral gene transfer and the fate of acquired genes

**DOI:** 10.1186/1471-2148-14-119

**Published:** 2014-06-05

**Authors:** Åke Strese, Anders Backlund, Cecilia Alsmark

**Affiliations:** 1 Division of Pharmacognosy, Department of Medicinal Chemistry, Uppsala University, Uppsala, Sweden; 2 Department of Virology, Immunobiology and Parasitology, National Veterinary Institute, Uppsala, Sweden

**Keywords:** Lateral gene transfer (LGT), *Trichomonas*, *Peptoniphilus*, Phylogeny

## Abstract

**Background:**

Lateral Gene Transfer (LGT) has recently gained recognition as an important contributor to some eukaryote proteomes, but the mechanisms of acquisition and fixation in eukaryotic genomes are still uncertain. A previously defined norm for LGTs in microbial eukaryotes states that the majority are genes involved in metabolism, the LGTs are typically localized one by one, surrounded by vertically inherited genes on the chromosome, and phylogenetics shows that a broad collection of bacterial lineages have contributed to the transferome.

**Results:**

A unique 34 kbp long fragment with 27 clustered genes (TvLF) of prokaryote origin was identified in the sequenced genome of the protozoan parasite *Trichomonas vaginalis*. Using a PCR based approach we confirmed the presence of the orthologous fragment in four additional *T. vaginalis* strains. Detailed sequence analyses unambiguously suggest that TvLF is the result of one single, recent LGT event. The proposed donor is a close relative to the firmicute bacterium *Peptoniphilus harei*. High nucleotide sequence similarity between *T. vaginalis* strains, as well as to *P. harei*, and the absence of homologs in other *Trichomonas* species, suggests that the transfer event took place after the radiation of the genus *Trichomonas*. Some genes have undergone pseudogenization and degradation, indicating that they may not be retained in the future. Functional annotations reveal that genes involved in informational processes are particularly prone to degradation.

**Conclusions:**

We conclude that, although the majority of eukaryote LGTs are single gene occurrences, they may be acquired in clusters of several genes that are subsequently cleansed of evolutionarily less advantageous genes.

## Background

The protozoan parasite *Trichomonas vaginalis* is a human pathogen that causes the most common, non-viral, sexually transmitted disease in the world, infecting 248 million people yearly according to WHO estimates [1]. Men are often asymptomatic carriers of the parasite, while symptoms in women range from malodorous vaginal discharge, inflammation and swelling of the urogenital tract to increased risk for cervical cancer, adverse pregnancy outcomes and an increased susceptibility to HIV-1 infection [2-4]. Treatment today is limited to two nitroimidazole derivatives, tinidazole and metronidazole, although failure of treatment due to resistance has been reported [5]. A draft genome sequence of *T. vaginalis* G3 was accomplished in 2007 [6], revealing an unusually large genome of more than 160 Mbp, encoding up to 60,000 genes in addition to numerous and diverse repeated regions.

LGT is the acquisition and fixation in the recipient genome of genetic material from a foreign donor organism without sexual transfer. It offers a rapid retrieval of new capabilities such as the ability to utilize new metabolites [7], degradation of chemicals such as pesticides [8] or the deployment of drug resistance genes [9]. The bacterial routes for uptake of foreign DNA are well described by features such as transformation, conjugation and transduction, or by the activities of “gene transfer agents” such as transposable elements. The mechanisms for eukaryotic gene acquisition are less well described [10], although one of the favored hypothesis suggests that the transfer is mediated via phagocytosis [11]. Recent finds, however, assume that e.g. transposable elements may facilitate uptake of prokaryote genes also in unicellular eukaryotes [12].

Laterally transferred genes have previously been identified with a phylogenomic approach in *T. vaginalis *[13,14], estimating that 0.25% of the genes in the genome were acquired by LGT from prokaryote or non-related eukaryote sources. These analyses in *T. vaginalis* and other protozoa also show that there are common features that hold true for the majority of genes acquired via LGT in unicellular eukaryotes [15]. One such feature is that LGTs are typically operational, rather than informational genes, a notion that has been predicted previously [16]. Another feature is that rather than one single bacterial donor lineage, as is the case for mitochondrial genes originating from an α-proteobacterial source, a broad range of prokaryotic donor organisms was identified. Furthermore, the LGTs in protozoa are typically not physically linked together on the recipient chromosome, but scattered between vertically inherited genes [17]. The haploid genome [14] of *T. vaginalis*, in combination with the assumption of an asexual reproduction [18], leaves this parasite with ample possibilities for fixation of acquired genes, since all genetic material within the nucleus is passed to the new generation following division [19]. Also, being unicellular, all new genetic material that is successfully incorporated in the genome of *T. vaginalis* will be passed on to all offspring following reproduction. In higher eukaryotes with multiple cells, however, a change in the genome must be fixated in a gamete in order to be passed on to its offspring.

In stark contrast to the observations of the norm for LGT in eukaryotes, the genome of *T. vaginalis* harbors a region of 34 kbp located on contig DS113827, at positions 18,333-52,058, that exhibits highly unusual characteristics. Our bioinformatic assessment based on similarity searches and phylogenetic analyses indicates that a recent transfer event may be the cause of this anomaly. This region, designated as the ***T****richomonas ****v****aginalis ***L**ateral gene transfer **F**ragment (TvLF), has been the target for in-depth analyses applying bioinformatic and molecular biology tools, with the purpose and aim to shed light on the comparably unknown features involved in the acquisition of LGT in eukaryotes.

## Results

### Confirming authenticity of TvLF as a part of the *T. vaginalis* genome

**Figure 1:**
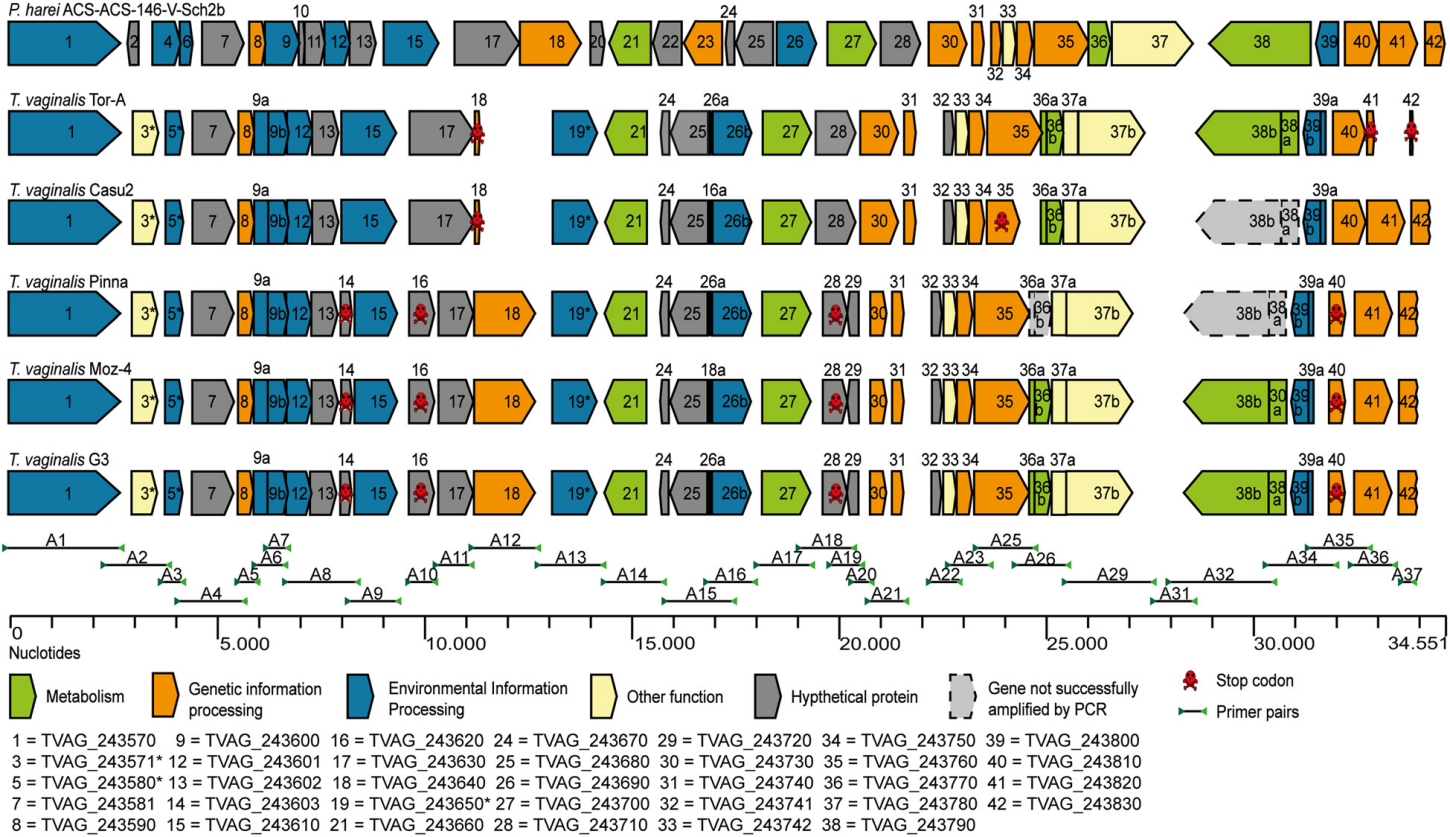
**Gene map overview of the genes of TvLF.** Overview of the genes of TvLF, in five strains investigated, of *T. vaginalis,* and the corresponding region in *P. harei*. Genes are numbered in order of appearance so that all orthologs have the same number. All details are listed in Additional file 1: Table S5. Note that *P. harei* contains eight genes without homologs in any strain of *T. vaginalis* (genes abbreviated 2, 4, 6, 10–11, 20, and 22–23) and *T. vaginalis* possess three genes absent in *P. harei* (genes denoted with asterisk, abbreviated 3, 5, and 19). Additionally, three genes (abbreviated 14, 16 and 29) are unique for *T. vaginalis* G3, Pinna and Moz-4, and are caused by stop codons in these strains. Gene classifications that are denoted by the different colors are according to Kyoto Encyclopedia of Genes and Genomes pathway (KEGG pathway). Genes without suitable KEGG-classification are categorized as “other function”. The majority of the primer-pairs used for amplifying and sequencing the genes of TvLF are visualized along with the primer-pair abbreviation found in Additional file 1: Table S8.

Compared to previously detected LGTs in parasites [13,20], the genes on TvLF have unusually high DNA sequence similarity to the putative donor *Peptoniphilus harei* (a firmicute bacterium), with no less than 79–98% identity at the amino acid level. An overview of the TvLF in comparison to the corresponding fragment in *P. harei* is visualized in the gene-map (Figure 1).

Such high identity levels are often considered to result from contaminants in the DNA source [21]. In this particular case, however, we present firm evidence providing an alternative explanation due to LGT. First, the genes of TvLF are not similar to known genes of *Mycoplasma,* a bacterium known to be frequently associated with, infect and multiply within *T. vaginalis* cultures [22-24]. Second, the *T. vaginalis* G3 strain used to sequence the complete genome was grown as an axenic culture [14]. Third, in addition to the putative LGTs the scaffold DS113827 containing the TvLF also encodes three consecutive genes, which are so far unique to *T. vaginalis*, TVAG_243540, TVAG_243550 and TVAG_243560. All three of these genes are repeated and dispersed also in other loci throughout the *T. vaginalis* G3 genome [14,25], as summarized in Additional file 1: Table S1.

To further establish that the genes on TvLF are genuinely part of the *T. vaginalis* genome, the physical connection between the *T. vaginalis*-specific repeated genes and the bacteria-like genes were re-confirmed by PCR and sequencing. During this process the size and nucleotide sequence of all annotated sequence gaps in the TvLF-region was determined (Additional file 1: Table S2). In addition, we have successfully amplified, using PCR, the proposed LGTs on TvLF in four additional strains of *T. vaginalis* (Table 1). Furthermore, the TvLF is present in the *T. vaginalis* G3 strain sequenced by the gene index project at TIGR. We consider the likelihood of the same contaminant affecting isolates maintained in three separate laboratories to be very small.

**Table 1:** **Identification of *T. vaginalis *strains used in this study**

**Strain**	**Isolated**	**Location**
G3 (PRA98)^1^	1973	Beckham, United Kingdom
Casu2 (SS-22)^1^	2008	Sardinia, Italy
Moz-4 (MPM4)^1^	1997	Mozambique
Pinna (SS-28)^1^	1998	Sardinia, Italy
Tor-A (TO-01)^1^	2010	Turin, Italy
T1^2^	1993	Taipei, Taiwan
P9^2^	-	Prague, Czech Republic

^1^Strains used in this study to investigate TvLF.

^2^Strains tested positive for the presence of three randomly selected TvLF genes.

However, to further rule out contamination, the presence of three randomly selected TvLF genes (TVAG_243650, 243760 and 243820) was confirmed in two additional strains (*T. vaginalis* T1 and *T. vaginalis* P9) that were not used for further sequence analysis. These seven isolates were maintained by three independent laboratories. Furthermore, PCR primers retrieved from the literature were used in an ultimately futile attempt to amplify regions of the 16 s-rDNA, to detect any bacterial contaminant in our sources of *T. vaginalis* DNA (Additional file 1: Table S3). Repeated attempts failed to amplify any products with primer pair 16 s:1, but pair 16 s:2 amplified the 18S ribosomal RNA gene of *T. vaginalis* (accession AY338475.1). No amplicons similar to any bacterial 16S rRNA genes were produced. In addition, fluorescence in situ hybridizations of probes within the TvLF to *T. vaginalis* nuclei confirm the presence of TvLF on one single locus in the *T. vaginalis* genome (Alsmark, unpublished data).

The TvLF appears to be specific to *T. vaginalis*, as none of the TvLF-genes could be identified by PCR in either *Trichomonas gallinae* or *Trichomonas tenax*, using primer sequences conserved in both *T. vaginalis* and *P. harei*. In previous studies *T. gallinae* and *T. tenax* have been verified to be the two most closely related species to *T. vaginalis* within the class of Trichomonadea [26]. This indicates that the transfer has occurred after the divergence of *T. vaginalis* from the remainder of the genus. A recent acquisition would be in agreement with the unusually high nucleotide sequence similarity to orthologs of the putative bacterial donor (Table 2).

**Table 2:** Genes of TvLF

**TvLF gene abbreviation**	**Annotated function**	**Top blastx hit**^**1**^	**Query coverage**	**Max identity**	**GenBank accession no.**
TVAG_243570	Cation transporting E1-E2 ATPase	*P. harei*	99%	96%	ZP_07822791.1
TVAG_243580	Transposase IS116/IS110/IS902 family protein	*S. pyogenes*	99%	94%	CAQ56293.1
TVAG_243590	Transcriptional regulator, gntR family protein	*P. harei*	99%	92%	ZP_07822817.1
TVAG_243600	ABC transporter	*P. harei*	99%	98%	ZP_07822813.1
TVAG_243610	Xanthine/uracil purine permease family protein	*P. harei*	99%	98%	ZP_07822771.1
TVAG_243620	Conserved hypothetical protein	*P. harei*	99%	90%	ZP_07822780.1
TVAG_243630	S-layer homology domain containing protein	*P. harei*	99%	94%	ZP_07822780.1
TVAG_243640	Glutamyl-tRNA synthetase	*P. harei*	99%	96%	ZP_07822745.1
TVAG_243650	Transposase family protein	*P.* sp*.*	49%	92%	ZP_07094114.1
TVAG_243660	Chitooligosaccharide deacetylase	*P. harei*	99%	92%	ZP_07822823.1
TVAG_243670	Conserved hypothetical protein	*P. harei*	98%	90%	ZP_07822757.1
TVAG_243680	Conserved hypothetical protein	*P. harei*	99%	91%	ZP_07822760.1
TVAG_243690	Auxin Efflux Carrier family protein	*P. harei*	99%	91%	ZP_07822772.1
TVAG_243700	S-adenosylmethionine synthetase	*P. harei*	99%	97%	ZP_07822781.1
TVAG_243710	Hypothetical protein	*P. harei*	85%	89%	ZP_07822767.1
TVAG_243720	Conserved hypothetical protein	*P. harei*	94%	99%	ZP_07822767.1
TVAG_243730	Conserved hypothetical protein	*P. harei*	89%	92%	ZP_07822775.1
TVAG_243740	DNA-binding protein HU	*P. harei*	62%	99%	ZP_07822787.1
TVAG_243750	S1 RNA binding domain containing protein, polyribonucleotide nucleotidyltransferase	*P. harei*	99%	95%	ZP_07822810.1
TVAG_243760	PP-loop family protein, cell cycle protein	*P. harei*	99%	90%	ZP_07822759.1
TVAG_243770	Hypoxanthine-guanine phosphoribosyltransferase	*P. harei*	99%	96%	ZP_07822797.1
TVAG_243780	Clan MA, family M41, FtsH endopeptidase-like metallopeptidase	*P. harei*	96%	96%	ZP_07822796.1
TVAG_243790	Penicillin binding protein transpeptidase,	*P. harei*	90%	97%	ZP_07822802.1
TVAG_243800	BioY family protein	*P. harei*	82%	93%	ZP_07822776.1
TVAG_243810	Conserved hypothetical protein (positive regulation of transcription, DNA-dependent)	*P. harei*	95%	99%	ZP_07822814.1
TVAG_243820	tRNA-dihydrouridine synthase	*P. harei*	99%	96%	ZP_07822761.1
TVAG_243830	Transcription elongation factor greA	*P. harei*	95%	97%	ZP_07822812.1

^**1**^*P. harei* = *Peptoniphilus harei. S. pyogenes* = *Streptococcus pyogenes. P. sp*. = *Peptoniphilus* species oral taxon 836.

### The genomic architecture of TvLF

The genes on TvLF encompass a stretch of 27 consecutive genes of bacterial origin, TVAG_243570-TVAG_243830, spanning more than 34 kbp of the 52 kbp long contig DS113827 in the *T. vaginalis* G3 genome (Figure 1, Table 2 and Additional file 1: Table S4 and Additional file 1: Table S5). Although absent from the sequenced eukaryote gene-pool, an homologous region was detected in the firmicute bacterium *Peptoniphilus harei* (contig 0004, positions 22397–56995, HMPREF9286_0330-HMPREF9286_0294, reverse direction). The TvLF stands in contrast to other LGTs detected in parasite genomes that typically are singletons embedded among vertically inherited genes [17,27].

A comprehensive comparative sequence analysis of the TvLF in *T. vaginalis* G3 and the putative bacterial donor reveals an unusually high degree of nucleotide sequence similarity (79-98%), compared to that of typical prokaryote-to-protozoa LGTs detected previously (27-83%) [14,17,28]. Comparing the gene order of TvLF with the corresponding region in *P. harei* also reveals long segments of synteny, another observation indicating that the transfer event was recent.

A PCR screen of the 27 TvLF genes in four additional strains of *T. vaginalis* yielded 105 out of the expected 108 PCR products. These 105 products were all sequenced on both strands. The inability to obtain an amplicon in the remaining three cases, despite numerous attempts, using both alternative primers designed in regions conserved between *T. vaginalis* and *P. harei* and exact match primers in sequences from PCR products in other strains, might be due to either actual gene loss, or to rearrangements resulting in loss of primer sites.

**Figure 2:**
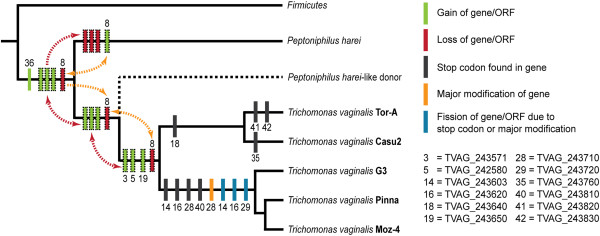
**Phylogenetic tree illustrating possible evolutionary events ultimately giving rise to TvLF.** Twenty-five genes and six non-annotated ORFs in the *T. vaginalis* G3 genome were acquired from a *P. harei*-like donor. Our hypothesis is that a close relative, extinct or not yet sequenced, to *P. harei* is the donor of the entire TvLF. Dotted arrows indicate alternative optimizations of the characters, and characters of uncertain position have dotted frames. For example, genes 3, 5 and 19 could have been acquired either by *T. vaginalis* after the LGT event or by the *P. harei*-like donor before the transfer. The three genes could also have been deleted in *P. harei* after the transfer event. Numbers above the bars indicate the count of events, while numbers below the bars refers to the gene abbreviation shown in Figure 2. (The same numbering is used in Figure 1 and in Additional file 1: Table S5, where a complete list of the genes is found).

Within the TvLF region, eight additional genes are encoded in *P. harei* but were not detected in any strain of *T. vaginalis*. These genes could have been acquired either by *P. harei* after the divergence of *P. harei* and the putative *P. harei*-like donor, or after the transfer of genes to *T. vaginalis*. Alternatively, the eight additional genes could have been present in the *P. harei*-like donor and transferred to *Trichomonas* but subsequently have been lost in the *T. vaginalis* genome (Figure 2, yellow dotted, arrowed lines).

Of the 27 TvLF genes, 23 show homology and are in synteny with genes in the corresponding region of *P. harei*, and are found in all strains investigated. Three of the remaining genes (TVAG_243571, TVAG_243580 and TVAG_243650, no. 3, 5 and 19 Figures 1 and 2) are found in all *T. vaginalis* strains but have not been identified in the complete genome sequence of *P. harei*. They do, however, exhibit high nucleotide sequence homology to sequences found in e.g. *P. rhinitidis* (WP_010242155.1) and *P.* str. F0141 (WP_009345232.1), as determined from phylogenetic analyses (Additional file 2: Figure S2). These LGTs (denoted by red dotted, arrowed lines) were either: 1) acquired by the common ancestor of *T. vaginalis* strains in an event distinct from the uptake of TvLF, 2) acquired by the *P. harei*-like donor and transferred along with the rest of the genes of TvLF to *Trichomonas*; or 3) lost in *P. harei* subsequent to the transfer event of TvLF to *T. vaginalis*. TVAG_243620 and TVAG_243720, (no. 16 and 29 in Figures 1 and 2) are only found in *T. vaginalis* strains G3, Moz-4 and Pinna, and are products of a gene fission caused by a stop codon and a major modification, respectively.

The comparison of TvLF genes annotated in *T. vaginalis* G3 with the homologous region in *P. harei* reveals 11 genes of equal length. In 7 of the remaining 16 genes, all of which are annotated as shorter in *Trichomonas* than in the bacterial orthologs, alternative start codons can be chosen to achieve longer ORFs that more closely resemble the *P. harei* orthologs (Additional file 1: Table S6).

In total we have detected eight TvLF genes that have accumulated stop codons: four genes in *T. vaginalis* strains G3, Pinna and Moz-4, and four distinct genes in *T. vaginalis* strains Tor-A and Casu2 (Table 3 and no. 14, 16, 18, 28, 35, 40–42 in Figures 1 and 2). For example, strains Tor-A and Casu2 have two cases of full-length genes equal to the corresponding genes in *P. harei*, while the two orthologs in *T. vaginalis* strains G3, Pinna, and Moz-4 are divided into four ORFs due to shared stop codons disrupting the genes (No. 16 and 17 as well as No. 28 and 29 respectively, in Figure 1). Similarly, TVAG_243610, together with the new ORF TVAG_243603 (No. 14 and 15 in Figure 1), are fragments in *T. vaginalis* G3, Pinna and Moz-4 corresponding to the beginning and end, respectively, of *P. harei* HMPREF9286_0319. However, this gene is intact in *T. vaginalis* Tor-A and Casu2. Mapping the termination codons causing this differential pseudogenization onto a phylogenetic tree shows that these events occur in a parsimonious fashion (Figure 2). Common to all genes of TvLF interrupted by termination codons is that they are all involved in information processes or annotated as hypothetical proteins. None of the genes involved in metabolic processes are found to have acquired stop codons.

**Table 3:** **TvLF genes in the five investigated *T. vaginalis *strains with acquired termination codons**

**Affected gene**	**Position**	**Strain**	**Comment**
TVAG_243610^2^	26605	G3, Pinna and Moz-4	Causes formation of TVAG_243603 and TVAG_243610
TVAG_243620^1,2^	28540	G3, Pinna and Moz-4	Causes formation of TVAG_243620 and TVAG_243630
TVAG_243640	30623	Tor-A and Casu2	
TVAG_243710^1,2^	38338	G3, Pinna and Moz-4	Involved in the formation of TVAG_243710 and TVAG_243720
TVAG_243760	42180	Casu2	
TVAG_243810^1^	50314	G3, Pinna and Moz-4	
TVAG_243820	50715	Tor-A	
TVAG_243830	51667	Tor-A	

^1^Termination codons annotated in *T. vaginalis* G3.

^2^Causes formation of *T. vaginalis* G3, Pinna and Moz-4 unique genes.

We have detected one major gene rearrangement (no. 28 in Figures 1 and 2) resulting in a unique gene, TVAG_243720, shared by *T. vaginalis* G3, Pinna and Moz-4. TVAG_243720 consists of the reversed end of the second half of the *P. harei* ortholog, immediately followed by a downstream deletion of approximately 400 bp. Within this 400 bp region, which still remains intact in *T. vaginalis* Tor-A and Casu2, an alternative start codon is present, which results in an extension of TVAG_243730 in *T. vaginalis* Tor-A and Casu2, which resemble the *P. harei* ortholog with respect to both size and nucleotide sequence similarity.

In addition to the 27 annotated TvLF genes, 7 previously un-annotated TvLF ORFs were identified by sequence comparison. Five of these ORFs have high nucleotide sequence similarity to genes of *P. harei*, while two ORFs are only present in other species of *Peptoniphilus*. These newly discovered ORFs were given names according to the gene directly upstream the ORF. Five of the seven new ORFs are present in all species and yield high scoring similarities to other genes, providing a tentative function (Additional file 1: Table S7). The two remaining ORFs (TVAG_243601 and TVAG_243602, no. 12 and 13 in Figure 1) contain conserved domain areas suggested by the Conserved Domain Database (CDD) to be ABC-2 family transporter proteins, and are only found in the strains of *T. vaginalis *[29].

Among the 27 genes of TvLF, eight are annotated as hypothetical proteins or genes involved in uncharacterized processes. Furthermore, four are categorized as involved in metabolic processes, eight as involved in genetic information processes, and seven in environmental information processes (transport). The latter two classes are both under-represented in other surveys of LGT in protozoa [13,30]. The pseudogenization is most widespread among genes involved in informational processes (five out of eight) as compared to those involved in transport (one of seven) and metabolism, where all genes are still intact (Table 4 and Additional file 1: Table S5).

**Table 4:** Functional distribution of LGTs in TvLF compared to the average functional distribution of LGTs in protists

**Gene function**	**No. genes in TvLF**	**No. pseudogenes in TvLF**	**% in TvLF**	**% Average in protists**^**a**^
Metabolism	4	0	14	44
Genetic information processing	8	5	28	6
Environmental information processing	7	1	24	3
Hypothetical	10	2	34	48

^a^[13].

### Phylogeny

In order to determine the potential donor of the genes of TvLF, as well as to investigate whether all genes may have one single donor, phylogenetic trees of all genes of TvLF were estimated. All trees (Additional file 2: Figure S2) support the close relationship of TvLF-sequences of *T. vaginalis* with bacteria of the Firmicute lineage, to the exclusion of any other bacterial or eukaryote sequence by at least one node with a minimum bootstrap support value of bs = 80%. All trees confirm the hypothesis of a close relative of *P. harei* as the likely donor organism. In two gene trees (TVAG_243650 and TVAG_243580), *T. vaginalis* clusters with other species of the *Peptoniphilus* lineage; however, in neither of these cases could orthologs be detected in the genome of *P. harei* using homology searches. This indicates that the donor organism is closely related, but not identical, to *P. harei*, and that this currently un-sequenced firmicute likely also possesses the orthologs to TVAG_243580 and 243650. Other possible, although from a parsimony perspective considerably less likely, scenarios include multiple donor organisms of the firmicute lineage or multiple losses in *P. harei*.

**Figure 3:**
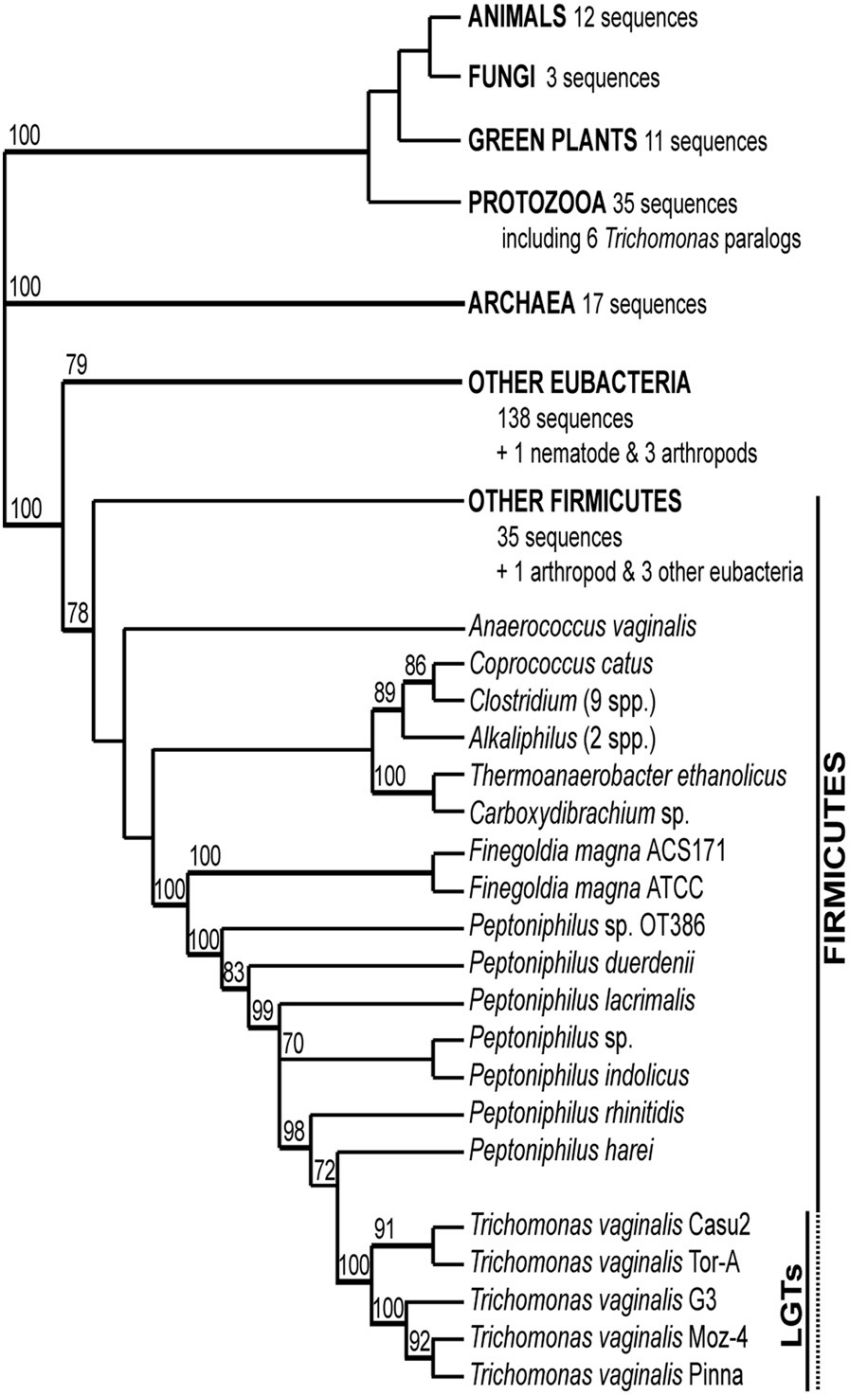
**Summarized synthetic phylogenetic tree of TvLF.** Synthetic phylogenetic tree summarizing congruent groupings retained from the analyses of separate gene support (Additional file 2: Figure S2) for the different TvLF gene homologs identified in various taxa, along with their corresponding bootstrap values. Large, well-supported clades with many representatives are reduced to placeholders, with the exception of the sections close to TvLF positions. All evaluated trees provide clear and unambiguous support for the placement of TvLF-sequences in the firmicute lineage.

To evaluate whether all genes were acquired in one single event a combined tree was produced by concatenating all sequences of TvLF and *P. harei* (with the exception of TVAG_243580 and TVAG_243650 that were coded as missing data in the analysis). The result is completely unambiguous and corroborates with high bootstrap support values the scenario shown in Figure 3.

The tree topology shows that *T. vaginalis* strains Tor-A and Casu2 form one group with high bootstrap support values (bs) from subsequent support analyses (bs = 91%), while *T. vaginalis* strains G3, Pinna and Moz-4 form another distinct and well supported clade (bs = 100%), in which the latter two also group together (bs = 92%). Thorough analysis of TvLF within these five different strains of *T. vaginalis* reveals a multitude of rearrangements, insertion, and deletion events (Figures 1 and 2), some of which render novel initiation or termination codons. Mapping the differences in TvLF between *T. vaginalis* strains on a phylogenetic sub-tree shows that all changes are parsimonious (Figure 2). This is a further evidence for the authenticity of TvLF as an integrated part of the *T. vaginalis* nuclear genome.

### Sequence analysis

**Figure 4:**
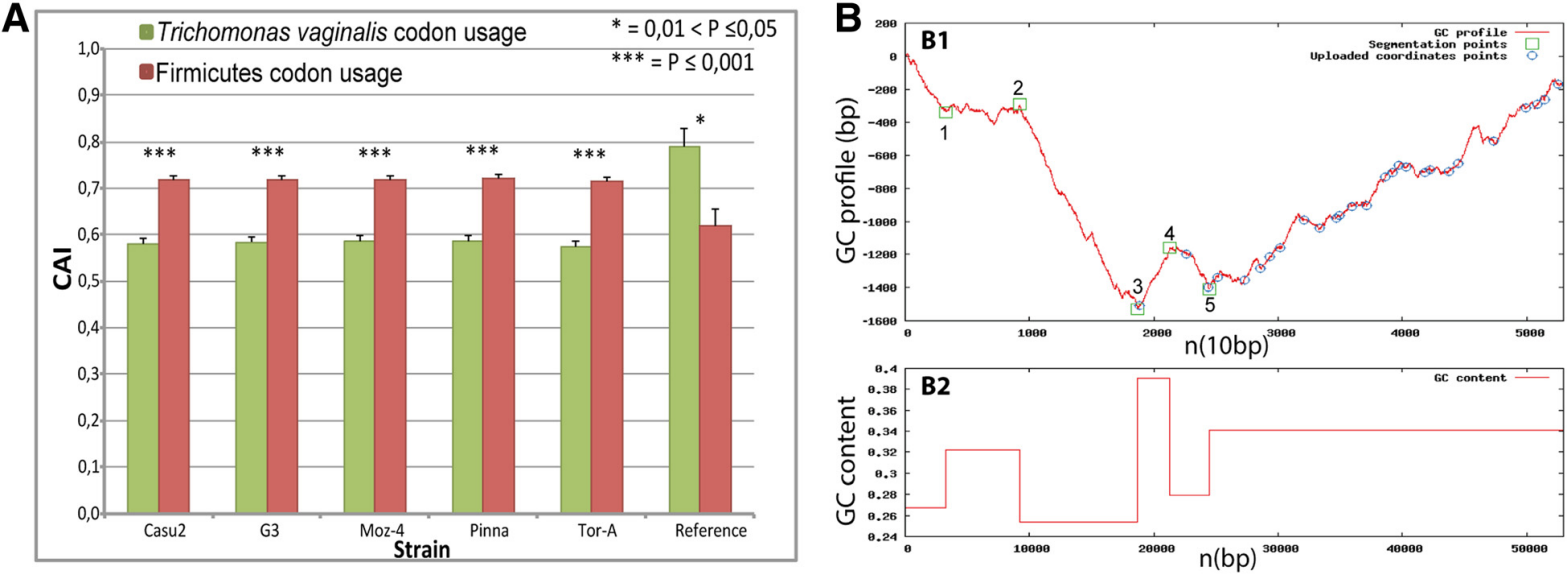
**The average Codon Adaptation index and the cumulative GC profile for TVLF. A**. The average Codon Adaptation Index (CAI) for each TvLF gene in the five *T. vaginalis* strains investigated. Green bars represent CAI calculated using the *T. vaginalis* codon usage data, and the red bars using firmicute codon usage data. Firmicute codon bias based CAI is significantly higher than *T. vaginalis* codon bias based CAI in all strains (p ≤ 0.0001). Furthermore, CAI calculated for the reference gene-set using firmicute codon bias is significantly lower than CAI calculated using *T. vaginalis* codon bias data (0.01 < P ≤ 0.05). A pooled, two sample t-test was used to determine significance. Error bars represent standard errors of the mean. **B1**. Negative z’ curve (cumulative GC profile) for TvLF *T. vaginalis* G3. Segmentation points are marked with green squares, and the positions of the bacteria-like genes are indicated with blue rings. Segmentation point 3 (segmentation strength 91.69) coincides with the beginning of the first LGT (the first *P. harei-*like gene, TVAG_243570). **B2**. The GC content distribution along the TvLF, using a 1000 bp sliding window.

CAI-values for the TvLF genes calculated with *T. vaginalis* codon usage data differ significantly from the values obtained for other firmicute codon usage data (Figure 4A). The average CAI for all genes of TvLF in each strain is significantly lower in *T. vaginalis* than in Firmicutes. This result is in line with the hypothesis that the TvLF was transferred recently, and that genetic information in the TvLF has not yet been molded to fit into the codon bias acting in *T. vaginalis*. The set of *T. vaginalis* housekeeping genes used as a reference has significantly higher CAI-value in *T. vaginalis.*

A cumulative GC profile was assembled to visualize the general nucleotide composition features of the TvLF. This has been suggested as a tool to indicate genomic islands or to identify LGTs [31,32]. The cumulative GC profile of scaffold DS113827 in the *T. vaginalis* G3 genome (Figure 4B) displays a strong segmentation (segmentation point 3, strength 90.48) at position 19,014 bp, with an increased GC content ending at position 21,591 bp. This segmentation point accords with the first *Peptoniphilus*-like gene, the first gene in TvLF, TVAG_243570. The strong segmentation point at 19,014 bp coinciding with the beginning of the TvLF further supports the hypothesis that this region was acquired from a foreign DNA source.

At the position of the second gene of TvLF, TVAG_243580, another segmentation point (point 4, strength 14.99) can be found with a small decrease in GC-content. This gene is not present in the genome of *P. harei*. However, in other species of *Peptoniphilus*, *P. indolicus* and *P. rhinitidis,* orthologs are retrieved with strong similarity scores at the DNA level and coherent positioning in the phylogenetic tree. These observations *could* indicate that the gene TVAG_243580, coding for a transposase, was acquired by LGT into the actual donor, as well as some of the other more distantly related *Peptoniphilus* species, after the divergence of the *Peptoniphilus* species but prior to the transfer-event giving rise to the TvLF. However, a more parsimonious view is that the gene was lost in *P. harei* but retained in the other species.

## Discussion

Our hypothesis is that a 34,102 bp long fragment, TvLF, encompassing 27 consecutive bacteria-like genes and seven un-annotated ORFs is the result of a recent gene transfer event from one single bacterial donor, presumably a close relative to the firmicute *P. harei*. This region stands in contrast to other LGTs detected in eukaryotic parasite genomes, which are almost always single gene occurrences embedded among genes of eukaryote origin [17,27].

The possibility that LGTs are initially acquired in clusters in protozoa has previously been proposed, although actual cases are rare. In *Giardia intestinalis* isolate GS three consecutive LGTs are found, although they are absent in *Giardia intestinalis* isolate WG, further advocating the hypothesis of a recent transfer [33]. In *Cryptosporidium* two separate pairs of genes appear to have been acquired at the same time [20]; and the ascomycete *Trichoderma* appears to have acquired a three-gene cluster resembling part of a nitrate assimilation pathway from a distantly related basidiomycete lineage [34]. Similar phenomena have also been observed in metazoan [35] and in plant mitochondria [36].

One reason for the apparent scattering of the typical LGTs in protozoa could be that once foreign DNA has been acquired and integrated into the host chromosome, there are two possible scenarios: loss or preservation. A newly acquired gene may degrade through mutational processes and vanish. If preserved, the LGT can relocate via internal recombination events, duplicate and evolve into a functional gene, possibly with a modified function [37,38]. This latter process, however, is as yet poorly understood, albeit the amelioration process has been shown to work rapidly on LGTs in bacteria, and would in these cases obliterate the compositional differences to the host genome [39]. Consequently, evaluation of compositional differences has been shown to be a poor marker for LGT [40].

The so-called “you are what you eat” hypothesis promoted by Doolittle (1998) suggests that genetic material can be incorporated into a unicellular eukaryotes genome. This may happen by chance after phagocytosis of bacteria populating the same habitat, and is supported by the fact that phagotrophs have a higher rate of LGT than non-phagotrophs [10,11]. Donors of LGTs in protozoa are predominantly bacteria sharing a habitat [13], making *Bacteroides*, *Clostridium*, and related species common contributors to *T. vaginalis*. Our findings support this theory, since *P. harei*, a firmicute and close relative of the assumed donor, shares a habitat with *T. vaginalis* in the urogenital tract and reproductive system of women [41-43]. We may further hypothesize that all of the LGTs in *T. vaginalis* that stem from, for example, the *Bacteroides* lineage may have been acquired in one or a few batches, in a similar mode to the TvLF, although genomic rearrangements in both donor and recipient have erased obvious evidence such as synteny.

Thus, we argue that the LGTs left in today’s protozoan genomes are the successfully fixed genes, still remaining after having passed evolutionarily driven rearrangements, and evaded gene decay. If the transfer of TvLF is as recent as indicated, it becomes reasonable to assume that this process is still ongoing, and that some genes may be retained under a selective pressure, while others evolve under more relaxed constraints and are likely to be lost in the future. In the case of TvLF we identify several instances where the latter situation occurs, for example genes disrupted by internal stop codons, resulting in pseudogenes.

Furthermore, if the physical uptake of genetic material is assumed to be random, while the fixation of genes is under a selective pressure [11,16], it follows that it is also reasonable that a very recent LGT-event has not yet been cleansed of obsolete material and streamlined to fit the exact requirements of the recipient organism. Such modifications have been shown to take place in bacteria during the amelioration process [39]. If we assume that similar processes are at work also in eukaryotes, then they have in TvLF presumably not yet had the time to homogenize the LGTs to resemble the remainder of the genome.

This is supported by results from the codon adaptation index (CAI) analyses for TvLF, where we show that CAI-values for genes in TvLF resemble those of the *P. harei* genome rather than those of the remainder of the *T. vaginalis* genome. The sequence similarity between the donor and the recipient is also higher than what is usually observed among LGTs. Similarly, calculation of cumulative GC values nominates the region between *T. vaginalis* specific repeated genes and TvLF, immediately adjacent to the locus of the first *Peptoniphilus*-like gene, as a site for incorporation of foreign DNA. Such strong segmentation points are described, for instance, in genomic islands found in bacteria with uniform GC-content [44,45].

The functional categorization of previously detected LGTs in bacteria and eukaryotes shows that most LGTs are active in metabolic processes, while informational genes are rare [11,13,16]. This phenomenon may reflect that, although the uptake happens by chance, the fixation does not, and that LGT predominantly is important for adaptation processes such as utilization of new metabolites, but less important for optimization of informational processes already encoded for by the cell.

In TvLF we have identified eight genes known to be active in genetic informational processes, such as transcription and cell cycle control; however, the majority of these have accumulated stop codons in one or more of the strains (five out of eight). This observation strengthens both the assumption that the TvLF region is evolving rapidly to remove undesirable genes and that the informational genes, to a certain extent, are within this less desirable category of genes. An additional observation that further confirms this hypothesis is that TvLF harbors *only* four genes known to be involved in metabolism, and all of these genes are intact. All four genes have previously been shown to be expressed [46], thus indicating that they may be functionally active.

More surprisingly, the TvLF encompass seven genes involved in transport, whereof six are intact in all of the strains investigated. A closer look at the functional annotation of these transport genes reveals that several are homologous to genes involved in antibiotic resistance in bacteria. Development of antibiotic resistance in bacteria is often achieved via LGT, but so far, to our knowledge, no such cases have been reported in protozoa. Whether some or all of these genes are actually active in *T. vaginalis* remains to be investigated.

Furthermore, in TvLF we have also observed two transposases, genes that are associated with transposition of genetic material, and may thus, hypothetically, be involved in the incorporation process during the actual uptake of TvLF. These transposases are not present in the corresponding region in *P. harei*, but are found in other Firmicutes of the *Peptoniphilus* lineage.

## Conclusions

In this study, the comprehensive comparative sequence analysis of the TvLF in five different strains of *Trichomonas* and the putative bacterial donor, *Peptoniphilus,* reveals an unusually high degree of nucleotide sequence similarity and synteny, supporting the hypothesis that TvLF is the result of a single, recent transfer event. Repeated attempts to amplify genes from the TvLF in other *Trichomonas* species, such as *T. gallinae* and *T. tenax*, have proven unsuccessful, indicating that the transfer occurred after the divergence of *T. vaginalis* from other trichomonads. Furthermore, an array of rearrangements, insertion, and deletion events – some of which render novel initiation or termination codons – are also found, indicating that part of TvLF is in a state of rapid evolutionary change. Mapping these features of TvLF onto a phylogenetic tree based on concatenated sequences of all genes in TvLF demonstrates that all of the features identified in this study can be explained in a parsimony framework.

Among the LGTs of TvLF, several are functionally annotated as genetic information processing genes, a functional category of genes that are under-represented among LGTs detected previously. However, most of these informational genes have been disrupted by internal stop codons. Uptake of long clusters of genes contributes a broad selection of novel functions to pick and choose from. The strategy to, at the same time, preserve the most beneficial genes only, may be the advantageous strategy to effectively gain new abilities.

Altogether, this study of the unique TvLF region demonstrates how the fixation process of recently acquired genetic material is shaped to resemble the norm for the microbial eukaryote transferome.

## Methods

### Organisms and cell culture

Four strains of *T. vaginalis* (*T. vaginalis* Casu2 SS22, *T. vaginalis* Moz-4 MPM4, *T. vaginalis* Tor-A TO 01, *T. vaginalis* Pinna SS28) were kindly provided by Dr. Pier Luigi Fiori at the Dept. of Biomedical Sciences, Division of Experimental and Clinical Microbiology, University of Sassari, Sassari, Italy; and *T. vaginalis* G3 DNA was provided from Prof. Robert Hirt at the Institute for Cell and Molecular Bioscience, Newcastle University. In addition, *T. vaginalis* T1 and P9, *T. gallinae* GCB P41 and *T. tenax* HS4 were provided by J. Tachezy, Department of Parasitology, Charles University, Prague, Czech Republic (Table 1). Cultures of trichomonads were grown as described previously [47]. DNA from all strains was extracted with DNeasy Blood and Tissue kit (Qiagen, Valencia, CA) and high Pure PCR Template Preparation Kit (Roche, Basel, CH) in accordance with the manufacturer’s instructions.

### PCR screen

PCR amplifications were performed in 50 μl reaction volume using either Mastercycler Personal (Eppendorf, Hamburg, D) or C1000™ Thermal Cycler (Bio-Rad, Hercules, CA): 75 mM Tris–HCl pH 8.5, 20 mM (NH_4_)_2_SO_4_, 2 mM MgCl_2_, 0.1% Tween 20®, 0.2 mM dNTPs and 1.25 U Ampliqon *Taq* polymerase (VWR International, Radnor, PN), 0,2-0,4 μM of each primer (Sigma-Aldrich, St. Louis, MO), 10–100 ng template and double distilled, sterile filtered using Milli-Q filter (Merck Millipor, Billerica, MA) water up to 50 μl. PCR amplification always began with one hold cycle at 95°C for 3 minutes followed by 30–35 cycles of 95°C for 30 s; primer melting point (Tm) minus 3–5°C for approximately 1 min/1000 bp product; and 72°C for 1 min. The amplification always ended with 72°C for 10 min as a final elongation step, followed by 4°C until manually shut down. Primers were designed in Geneious v.5 and v.6 (Biomatters, Auckland, NZ) using the Primer3 algorithm [48,49]. Primer specification can be found in Additional file 1: Table S8.

PCR products were analyzed on-chip with a MultiNA (Shimadzu, Kyoto, J); and positive amplifications were purified with GenElute™ PCR Clean-Up kit (Sigma-Aldrich), sequenced through Standard-Seq by Macrogen (Macrogen Corporation, Amsterdam, NL) and assembled using Geneious. All assemblies were based on sequences from both strands with a minimum of two-fold coverage. Measures to avoid errors consisted of by eye examination of assemblies, before consensus sequences were deposited in GenBank.

### Sequence analysis

A comprehensive search for potential un-annotated ORFs (open reading frames) and other sequence ambiguities in the entire scaffold DS113827 was performed and compared to the corresponding region of *P. harei*, using Geneious and National Center for Biotechnology Information (NCBI) Basic Local Alignment Search Tool, BLAST [50].

A cumulative GC profile was assembled to visualize the general composition features of the TvLF using GC-content data [31]. Input parameters were set to the default, with the exception of the ‘halting parameter’, which was adjusted to 10. In the GC plot a coordinate file was incorporated consisting of *T. vaginalis* G3 LGTs positions, to visualize the correlation between the GC-shifts and the spatial distribution of the bacteria-like genes.

The codon adaptation index (CAI) is suggested as a way to measure synonymous codon usage bias [51,52]. *T. vaginalis* has been suggested to have a biased codon usage, and therefore becomes suitable for CAI calculation [53]. CAI estimates have also been used to evaluate whether the genetic material is recently acquired, as the host should not have had time to adapt recently retrieved material to fit its own codon usage [54]. In this particular case, however, the time for acquisition will be identical for all genes in TvLF, and thus this aspect becomes less important.

CAI were calculated for each TvLF gene for all *T. vaginalis* strains using the web based CAI-Calculator by Puigbo and co-workers [55]. Also, a set of four *T. vaginalis* housekeeping genes was used as reference: TVAG_343390: mannose-6-phosphate isomerase; TVAG_299450: alanyl-tRNA synthetase; TVAG_258340: family T2 asparaginase-like threonine peptidase; and TVAG_054490: tryptophanase [56]. As codon usage data, the codon usage table for *T. vaginalis* based on 189 CDSs (65,401 codons) was employed. Because the absence of a *P. harei* codon usage table, two other closely related (Figure 3) firmicute bacteria codon usage tables, *Clostridium thermocellum* ATCC 27405 based on 3191 CDS’s (1,072,649 codons) and *Lactobacillus gasseri* ATCC 33323 based on 1755 CDSs (558,761 codons) served as template to calculate CAI and the average CAI-values from the two different bacteria were used. The codon usage tables were acquired from public online resources (http://www.kazusa.or.jp/codon/).

In order to evaluate the evolutionary adaptation, a comparison was made between the average CAI-values on the TvLF genes calculated with *T. vaginalis* codon usage and the average CAI-values obtained from calculations made with both *Clostridium thermocellum* and *Lactobacillus gasseri*.

### Phylogenetic analyses

Phylogenetic analyses of the different genes in TvLF were executed in order to determine their evolutionary origin. TvLF genes were used as the query sequence to perform homology searches using the blastx-algorithm [50] on the non-redundant protein sequences (NR) database (http://blast.ncbi.nlm.nih.gov/Blast), and the relevant homologs were collected. The matrices aimed to include homologs from five firmicute bacteria, 20 bacteria from other phyla, and 20 eukaryotic organisms of different phyla.

Alignments were performed using ClustalW algorithm [57] and subsequently inspected and edited manually using Geneious. The phylogenetic analyses of the obtained matrices were performed using PAUP* v. 4.0b10 software (Sinauer Assoc. Inc., Sunderland, MA; [58] running under Mac OS X on an MacBook Pro with 8 GB of memory available, SSD and 3.8 GHz Intel Core i7 processor (Apple, Cupertino, CA). The analyses were executed under the maximum parsimony criterion [59], and included 1,000 random addition sequence replicates followed by TBR branch-swapping, followed by the calculation of a strict consensus tree. Tree support was estimated using a bootstrap approach [60] with 100 bootstrap replicates, each followed by 100 random addition sequence replicates, followed by SPR branch-swapping. All strict consensus trees and general analysis statistics are supplied in Additional file 2: Figure S2.

### Data access

Generated data are uploaded to GenBank and were allocated the following accession numbers; KF269355-KF269530. http://www.ncbi.nlm.nih.gov/nuccore/KF269355.1 - http://www.ncbi.nlm.nih.gov/nuccore/KF269530.1.

The data set supporting the results of this article is available in the Dryad repository; http://datadryad.org/resource/doi:10.5061/dryad.30r6p [61].

## Competing interests

The authors declare that they have no competing interests.

## Authors’ contributions

ÅS performed the experimental work and the bulk of the bioinformatic analyses, complemented by analyses from CA and AB. CA conceived and coordinated the project. All authors wrote and edited the manuscript. All authors read and approved the final manuscript.

## Supplementary Material

Additional file 1: Table S1 Listing the *Trichomonas* unique genes paralogs located on the DS113827 contig, directly adjacent to the TvLF. **Table S2.** Contains information regarding the annotated gaps in contig DS113827. **Table S3.** Lists the 16 s rDNA primers used for detection of posible contaminant bactera. **Table S4.** Contains detailed information on extended ORFS in TvLF; and **Table S5.** Has a detailed list of the genes of TvLF. **Table S6.** Containes a list with abbreviations found in the gene map (Figure 1). **Table S7.** Contains data on newly discovered ORFs in TVLF. **Table S8.** Lists the oligonucleotides used in this study. Click here for file

Additional file 2: Figure S2 Summary and results from phylogenetic analyses of all 27 genes of the TVLF, and analysis to elucidate relationship between *Trichomonas vaginalis* strains. Click here for file
