# Supplementary material for: A recently transferred cluster of bacterial genes in Trichomonas vaginalis - lateral gene transfer and the fate of acquired genes
Source: BMC Evol Biol. 2014 Jun 5;14:119. doi: 10.1186/1471-2148-14-119 (PMC4082486; doi:10.1186/1471-2148-14-119)
Supplement: Additional file 2: Figure S2 — Summary and results from phylogenetic analyses of all 27 genes of the TVLF, and analysis to elucidate relationship between Trichomonas vaginalis strains. [file 1471-2148-14-119-S2.docx]

**Figure S2: TvLF genes strict consensus trees and general statistics.**

1. TVAG_243570.nxs

/––––––––––––––––––––––––––––––––––––––––––––––––––––––––––––––––– Crassostrea gigas

| /––––––––– Desulfurococcus kamchatkensis

+––––––––––––––––––––––––––56–––––––––––––––––––––––––––+––––––––– Methanosarcina mazei

+––––––––––––––––––––––––––––––––––––––––––––––––––––––––––––––––– Methanosaeta concilii

+––––––––––––––––––––––––––––––––––––––––––––––––––––––––––––––––– Truepera radiovictrix

+––––––––––––––––––––––––––––––––––––––––––––––––––––––––––––––––– Sulfurovum sp.

| /––––––––––––––––––– Nitrosospira multiformis

| /––100–––+ /––––––––– Nitrobacter sp.

+–––––––––––––––––60–––––––––––––––––+ \–––90––––+––––––––– Starkeya novella

| \–––––––––––––––––––––––––––– Caldilinea aerophila

| /––––––––––––––––––– Cyanothece sp.

+–––––––––––––––––––––60––––––––––––––––––––––+ /––––––––– Syntrophus aciditrophicus

/–––100–––+ \–––99––––+––––––––– Thiobacillus denitrificans

| | /––––––––– Collinsella tanakaei

| | /–––56––––+––––––––– Enterococcus faecalis

| | +––––––––––––––––––– Acholeplasma laidlawii

| | | /––––––––– Sphaerochaeta coccoides

| +–––––––––––––––––––––66––––––––––––––––––––––+–––100–––+––––––––– Sphaerochaeta pleomorpha

| | +––––––––––––––––––– Sebaldella termitidis

| | \––––––––––––––––––– Petrotoga mobilis

| +––––––––––––––––––––––––––––––––––––––––––––––––––––––––––––––––– Anaerolinea thermophila

| |

/––––––––+ | /–––––––––––––––––––––––––––––––––––––––––––––––––––––––– Helcococcus kunzii

| | | | /––––––––––––––––––––––––––––––––––––––––––––––– Finegoldia magna

| | \––**100**–––+ | /––––––––––––––––––––––––––––––––––––– Peptoniphilus rhinitidis

| | \–––61–––+ | /–––––––––––––––––––––––––––– Peptoniphilus harei

| | \–––**100**–––+ | /––––––––– Trichomonas vaginalis Pinna

| | \––**100**–––+ /–––62––––+––––––––– Trichomonas vaginalis G3

| | \––100–––+ \––––––––– Trichomonas vaginalis Moz–4

| | +––––––––––––––––––– Trichomonas vaginalis Casu2

| | \––––––––––––––––––– Trichomonas vaginalis Tor–A

| |

| \––––––––––––––––––––––––––––––––––––––––––––––––––––––––––––––––––––––––––– Monosiga brevicollis

\–––––––––––––––––––––––––––––––––––––––––––––––––––––––––––––––––––––––––––––––––––– Danio rerio

Trichomonas – Firmicutes – Bacteria – Eukaryota – Animalia – Plantae – Archaea **designative nodes**

1. TVAG_243580.nxs

/––––––––––––––––––––––––––––––––––––––––––––––––––––––––––––––––––– Fusobacterium nucleatum

| /––––––––––– Hydrogenivirga sp.

| /–––––––––––––––––––––74–––––––––––––––––––––+––––––––––– Thermodesulfovibrio yellowstonii

/––––91––––+ +–––––––––––––––––––––––––––––––––––––––––––––––––––––––– Sulfurihydrogenibium sp.

| | |

| | | /––––––––––––––––––––––––––––––––––––––––––––– Clostridium scindens

| \––––71––––+ | /–––––––––––––––––––––– Streptococcus pyogenes

| | | | /––––––––––– Trichomonas vaginalis Casu2

| | | | +––––––––––– Trichomonas vaginalis G3

| \––––**93**––––+ +––––87––––+––––––––––– Trichomonas vaginalis Moz-4

/––––––––––+ | /––––55––––+ +––––––––––– Trichomonas vaginalis Pinna

| | | | | \––––––––––– Trichomonas vaginalis Tor-A

| | | | +–––––––––––––––––––––– Peptoniphilus rhinitidis

| | \––––**99**–––––+ \–––––––––––––––––––––– Finegoldia magna

| | | /––––––––––– Peptoniphilus indolicus

| | \–––––––––87––––––––––+––––––––––– Anaerococcus prevotii

| |

| \–––––––––––––––––––––––––––––––––––––––––––––––––––––––––––––––––––––––––––––– Ktedonobacter racemifer

\––––––––––––––––––––––––––––––––––––––––––––––––––––––––––––––––––––––––––––––––––––––––– Gluconacetobacter europaeus

Trichomonas – Firmicutes – Bacteria – Eukaryota – Animalia – Plantae – Archaea **designative nodes**

1. TVAG_243590.nxs

/––––––––––––––––––––––––––––––––––––––––––––––––––––––––––––––––––––––––––––––––––––– Marinitoga piezophila

| /–––––––––––––––––––––––––––––––––––––––––––––––––––––––––––––––– Treponema phagedenis

| | /––––––––––––––––––––– Pseudoxanthomonas suwonensis

| +––––––––––––––––––––80––––––––––––––––––––+––––––––––––––––––––– Asticcacaulis biprosthecum

| | \––––––––––––––––––––– Herpetosiphon aurantiacus

| +–––––––––––––––––––––––––––––––––––––––––––––––––––––––––––––––– Collinsella aerofaciens

| |

| | /––––––––––––––––––––––––––––––––––––––––––– Peptoniphilus harei

| | | /––––––––––––––––––––– TVAG 243590 Casu2

| +––––––––**100**–––––––––+ +––––––––––––––––––––– TVAG 243590 G3

| | \–––––––––95––––––––––+––––––––––––––––––––– TVAG 243590 Pinna

\––––––––––––––––––––+ +––––––––––––––––––––– TVAG 243590 Moz–4

| \––––––––––––––––––––– TVAG 243590 Tor–A

|

+–––––––––––––––––––––––––––––––––––––––––––––––––––––––––––––––– Faecalibacterium prausnitzii

+–––––––––––––––––––––––––––––––––––––––––––––––––––––––––––––––– Clostridium sp.

+–––––––––––––––––––––––––––––––––––––––––––––––––––––––––––––––– Flavonifractor plautii

| /––––––––––––––––––––– Sporosarcina newyorkensis

+––––––––––––––––––––57––––––––––––––––––––+––––––––––––––––––––– Psychroflexus torquis

+–––––––––––––––––––––––––––––––––––––––––––––––––––––––––––––––– Mobiluncus mulieris

+–––––––––––––––––––––––––––––––––––––––––––––––––––––––––––––––– Rhodopirellula baltica

| /––––––––––––––––––––– Bacteroides sp.

+–––––––––––––––––––100––––––––––––––––––––+––––––––––––––––––––– Parabacteroides johnsonii

\–––––––––––––––––––––––––––––––––––––––––––––––––––––––––––––––– Methanocorpusculum labreanum

Trichomonas – Firmicutes – Bacteria – Eukaryota – Animalia – Plantae – Archaea **designative nodes**

1. TVAG_243600.nxs

/––––––––––––––––––––––––––––––––––––––––––––––––––––––––––––––––––––––––––––––––––––– Thermotoga sp.

| /–––––––––––––––––––––––––––––––––––––––––––––––––––––––––––––––––––– Halobacterium sp.

| | /––––––––––––––––– Pseudoalteromonas sp.

| | /–––––––––––––––83––––––––––––––––+––––––––––––––––– Schlesneria paludicola

\––––––––––––––––+ | /–––––––––––––––––––––––––––––––––– Treponema brennaborense

| | +–––––––––––––––––––––––––––––––––– Sphaerochaeta pleomorpha

\–––––––94–––––––+ +–––––––––––––––––––––––––––––––––– Eggerthella sp.

| +–––––––79–––––––+––––––––––––––––– Psychroflexus torquis

| | \––––––––––––––––– Paenibacillus sp. HGF5

| |

| | /––––––––––––––––– Peptoniphilus harei

| | +––––––––––––––––– Trichomonas vaginalis G3

| | +––––––––––––––––– Trichomonas vaginalis Moz–4

\–––––––62–––––––+––––––**100**–––––––+––––––––––––––––– Trichomonas vaginalis Casu2

| +––––––––––––––––– Trichomonas vaginalis Tor–A

| \––––––––––––––––– Trichomonas vaginalis Pinna

|

+–––––––––––––––––––––––––––––––––– Clostridiales bacterium

| /––––––––––––––––– Coprococcus catus

+––––––100–––––––+––––––––––––––––– Eubacterium ventriosum

+–––––––––––––––––––––––––––––––––– Acetobacterium woodii

\–––––––––––––––––––––––––––––––––– Methanocorpusculum labreanum

Trichomonas – Firmicutes – Bacteria – Eukaryota – Animalia – Plantae – Archaea **designative nodes**

1. TVAG_243610.nxs

/––––––––––––––––––––––––––––––––––––––––––––––––––––––––––––––––––––––––––––––––––––– Homo sapiens

| /–––––––––––––––––––––––––––––––––––––––––––––––––––––––––––––––––––––––––– Methanococcus maripaludis

| |–––––––––––––––––––––––––––––––––––––––––––––––––––––––––––––––––––––––––– Vibrio furnissii

| | /–––––––––––––––––––––––––––––––– Solitalea canadensis

| | +–––––––––––––––––––––––––––––––– Spirochaeta smaragdinae

| | /––––––––––––––54–––––––––––––––+ /–––100–––+––––––––––– Marinilabilia salmonicolor

| | | \––––65––––+ \––––––––––– Anaerophaga thermohalophila

| | | \––––––––––––––––––––– Alkaliphilus metalliredigens

| | |

| | | /–––––––––––––––––––––––––––––––– Peptoniphilus harei

| | | | /––––––––––––––––––––– Trichomonas vaginalis G3

| +–––87––––+ /–––**100**––––+ +––––––––––––––––––––– Trichomonas vaginalis Moz–4

\––––––––––+ | | \––––86––––+––––––––––––––––––––– Trichomonas vaginalis Pinna

| | /–––56––––+ | /––––––––––– Trichomonas vaginalis Casu2

| | | | \–––84––––+––––––––––– Trichomonas vaginalis Tor–A

| | | |

| \––––98––––+ \––––––––––––––––––––––––––––––––––––––––––– Finegoldia magna

| \––––––––––––––––––––––––––––––––––––––––––––––––––––– Anaerococcus vaginalis

| /–––––––––––––––––––––––––––––––– Populus trichocarpa

| | /––––––––––– Malassezia globosa

\–––––––––––––––––––66––––––––––––––––––––+ /–––69––––+––––––––––– Physcomitrella patens

\–––100––––+––––––––––––––––––––– Thalassiosira oceanica

Trichomonas – Firmicutes – Bacteria – Eukaryota – Animalia – Plantae – Archaea **designative nodes**

1. TVAG_243620-243630.nxs

/–––––––––––– Trichomonas vaginalis Pinna

/––––99––––+–––––––––––– Trichomonas vaginalis Moz-4

| \–––––––––––– Trichomonas vaginalis G3

/––––74–––––+––––––––––––––––––––––– Trichomonas vaginalis Tor-A

/––––**100**––––+ \––––––––––––––––––––––– Trichomonas vaginalis Casu2

/––––**100**––––+ \––––––––––––––––––––––––––––––––––– Peptoniphilus harei

| \––––––––––––––––––––––––––––––––––––––––––––––– Peptoniphilus rhinitidis

/––––61––––+ /–––––––––––– Peptoniphilus sp oral taxon 836

/–––––––––––+ \–––––––––––––––––––––100––––––––––––––––––––––+–––––––––––– Peptoniphilus lacrimalis

| \–––––––––––––––––––––––––––––––––––––––––––––––––––––––––––––––––––––– Peptoniphilus duerdenii

\–––––––––––––––––––––––––––––––––––––––––––––––––––––––––––––––––––––––––––––––––– Peptoniphilus indolicus

Trichomonas – Firmicutes – Bacteria – Eukaryota – Animalia – Plantae – Archaea **designative nodes**

1. TVAG_243640.nxs

/––––––––––––––––––––––––––––––––––––––––––––––––––––––––––––––––––––––––––– Crassostrea gigas

| /––––––––– Branchiostoma floridae

| /–––––––––––––––––––––––––––54–––––––––––––––––––––––––––+––––––––– Ixodes scapularis

| | /–––––––––––––––––––––––––––––––––––––––––––––––––––––––– Wickerhamomyces ciferrii

| | +–––––––––––––––––––––––––––––––––––––––––––––––––––––––– Capsaspora owczarzaki

| | +–––––––––––––––––––––––––––––––––––––––––––––––––––––––– Puccinia graminis

| | +–––––––––––––––––––––––––––––––––––––––––––––––––––––––– Rhizopus delemar

| | +–––––––––––––––––––––––––––––––––––––––––––––––––––––––– Phytophthora infestans

| | +–––––––––––––––––––––––––––––––––––––––––––––––––––––––– Dictyostelium purpureum

| | | /––––––––– Glycine max

| | +––––––––––––––––––––––76––––––––––––––––––––––+––––––––– Brachyspira murdochii

| | | /––––––––– Fusobacterium sp.

\––––––––+ +–––––––––––––––––––––100––––––––––––––––––––––+––––––––– Leptotrichia goodfellowii

| +–––––––––––––––––––––––––––––––––––––––––––––––––––––––– SAR324 cluster bacterium JCVISC AAA005(13)

| | /––––––––––––––––––– Rhodothermus marinus

\–––93––––+ +––––––––––––––––––– Melioribacter roseus

+–––––––––––––––––52–––––––––––––––––+ /––––––––– Chlorobium chlorochromatii

| \–––97––––+––––––––– Chloroherpeton thalassium

|

| /––––––––– Finegoldia magna ACS171

| /–––––––––––––––––100–––––––––––––––––+––––––––– Finegoldia magna ATCC

| | /–––––––––––––––––––––––––––––––––––––– Peptoniphilus duerdenii

\–––**99**–––+ | /–––––––––––––––––––––––––––– Peptoniphilus rhinitidis

\–––72–––+ | /––––––––––––––––––– Peptoniphilus harei

\–––**100**–––+ | /––––––––– Trichomonas vaginalis Casu2

\––**100**–––+ +––––––––– Trichomonas vaginalis G3

\–––100–––+––––––––– Trichomonas vaginalis Moz–4

+––––––––– Trichomonas vaginalis Pinna

\––––––––– Trichomonas vaginalis Tor–A

Trichomonas – Firmicutes – Bacteria – Eukaryota – Animalia – Plantae – Archaea **designative nodes**

1. TVAG_243650.nxs

/–––––––––––––––––––––––––––––––––––––––––––––––––––––––––––––––––––––––––––––– Microcystis aeruginosa

| /–––––––––––––––––––––––––––––––––––––––––––––––––––––––––––––––––––– Acinetobacter sp.

| | /––––––––––––––––––––––––––––––––––––––––––––––––––––––––––– Fusobacterium periodonticum

\–––––––––+ | /––––––––––––––––––––––––––––––––––––––––––––––––– Peptoniphilus rhinitidis

\–––60–––+ | /–––––––––– Eubacterium saburreum

| | /–––69––––+–––––––––– Clostridiales bacterium

\–––91––––+ /––––––––60––––––––+ /–––––––––– Caldicellulosiruptor kronotskyensis

| | \–––100–––+–––––––––– Fervidobacterium nodosum

| |

| | /–––––––––– Trichomonas vaginalis Tor-A

\–––64––––+ /–––65––––+–––––––––– Trichomonas vaginalis Casu2

| +–––––––––––––––––––– Trichomonas vaginalis G3

| /–––95–––+–––––––––––––––––––– Trichomonas vaginalis Moz-4

\–––**100**–––+ \–––––––––––––––––––– Trichomonas vaginalis Pinna

\––––––––––––––––––––––––––––– Peptoniphilus str.

Trichomonas – Firmicutes – Bacteria – Eukaryota – Animalia – Plantae – Archaea **designative nodes**

1. TVAG_243660.nxs

/–––––––––––––––––––––––––––––––––––––––––––––––––––––––––––––––––––––––––––––––––––––– Leptotrichia buccalis

| /–––––––––––––––––––––––––––––––––––––––––––––––––––––––––––––––––––––––––– Sebaldella termitidis

| | /–––––––––––– Methylobacter tundripaludum

| | /–––––––––––––––––––––––57–––––––––––––––––––––––+–––––––––––– Microcoleus chthonoplastes

| | +––––––––––––––––––––––––––––––––––––––––––––––––––––––––––––– Bradyrhizobium sp.

\–––––––––––+ +––––––––––––––––––––––––––––––––––––––––––––––––––––––––––––– Oxalobacter formigenes

| | /––––––––––––––––––––––––––––––––––––––––––––––––– Propionibacterium avidum

| | +––––––––––––––––––––––––––––––––––––––––––––––––– Enterococcus faecalis

\–––––99–––––+ +––––––––––––––––––––––––––––––––––––––––––––––––– Filifactor alocis

| +––––––––––––––––––––––––––––––––––––––––––––––––– Streptococcus mutans

| |

| | /––––––––––––––––––––––––––––––––––––– Peptoniphilus harei

\––––72–––––+ | /––––––––––––––––––––––––– Trichomonas vaginalis Casu2

+––––**100**––––+ +––––––––––––––––––––––––– Trichomonas vaginalis Tor-A

| \––––99–––––+ /–––––––––––– Trichomonas vaginalis G3

| \–––––64–––––+–––––––––––– Trichomonas vaginalis Moz-4

| \–––––––––––– Trichomonas vaginalis Pinna

|

+––––––––––––––––––––––––––––––––––––––––––––––––– Finegoldia magna

\––––––––––––––––––––––––––––––––––––––––––––––––– Parvimonas sp.

Trichomonas – Firmicutes – Bacteria – Eukaryota – Animalia – Plantae – Archaea **designative nodes**

1. TVAG_243670.nxs

/––––––––––––––––––––––––––––––––––––––––––––––– Planctomycetes KSU1

+––––––––––––––––––––––––––––––––––––––––––––––– Geobacter sulfurreducens

+––––––––––––––––––––––––––––––––––––––––––––––– Synergistetes bacterium

+––––––––––––––––––––––––––––––––––––––––––––––– Brachyspira pilosicoli

+––––––––––––––––––––––––––––––––––––––––––––––– Leptotrichia buccalis

+––––––––––––––––––––––––––––––––––––––––––––––– Pyramidobacter piscolens

+––––––––––––––––––––––––––––––––––––––––––––––– Desulfurispirillum indicum

+––––––––––––––––––––––––––––––––––––––––––––––– Jonquetella anthropi

+––––––––––––––––––––––––––––––––––––––––––––––– Slackia exigua

/––––99–––––+––––––––––––––––––––––––––––––––––––––––––––––– Anaerococcus vaginalis

| |

| | /–––––––––––––––––––––––––––––––––––– Peptoniphilus indolicus

| | | /–––––––––––––––––––––––– Peptoniphilus harei

| +––––66––––+ | /–––––––––––– Trichomonas vaginalis Casu2

| | \––––**93**–––––+ +–––––––––––– Trichomonas vaginalis G3

| | \––––98–––––+–––––––––––– Trichomonas vaginalis Moz-4

/–––––––––––+ | +–––––––––––– Trichomonas vaginalis Pinna

| | | \–––––––––––– Trichomonas vaginalis Tor-A

| | |

| | | /–––––––––––––––––––––––– Succinivibrionaceae bacterium

| | \––––––––––57––––––––––+ /–––––––––––– Oribacterium sp.

| | \––––92–––––+–––––––––––– Oribacterium sp.

| \––––––––––––––––––––––––––––––––––––––––––––––––––––––––––– Bradyrhizobium sp.

\––––––––––––––––––––––––––––––––––––––––––––––––––––––––––––––––––––––– Rhodopseudomonas palustris

Trichomonas – Firmicutes – Bacteria – Eukaryota – Animalia – Plantae – Archaea **designative nodes**

1. TVAG_243680.nxs

/––––––––––––––––––––––––––––––––––––––––––––––––––––––––––––––––––––––––––––––––––––– Peptoniphilus rhinitidis

| /––––––––––––––––––––––––––––––––––––––––––– Peptoniphilus harei

| | /––––––––––––––––––––– Trichomonas vaginalis Casu2

| /––––––––**100**–––––––––+ +––––––––––––––––––––– Trichomonas vaginalis G3

| | \–––––––––100–––––––––+––––––––––––––––––––– Trichomonas vaginalis Moz-4

| | +––––––––––––––––––––– Trichomonas vaginalis Pinna

\––––––––––––––––––––+ \––––––––––––––––––––– Trichomonas vaginalis Tor-A

| /––––––––––––––––––––– Peptoniphilus indolicus

\–––––––––––––––––––100––––––––––––––––––––+––––––––––––––––––––– Peptoniphilus sp.

Trichomonas – Firmicutes – Bacteria – Eukaryota – Animalia – Plantae – Archaea **designative nodes**

1. TVAG_243690.nxs

/–––––––––––––––––––––––––––––––––––––––––––––––––––––––––––––––––––– Methanocella paludicola

| /––––––––––––––––––––––––––––––––––––––––––––––––––––––––– Haloferax volcanii

| | /––––––––––– Fusobacterium nucleatum

| | /–––––––––––––––82––––––––––––––––+––––––––––– Treponema vincentii

\––––––––––+ | /–––––––––––––––––––––––––––––––––– Ignavibacterium album

| +––––64––––+ /––––––––––––––––––––––– Photobacterium profundum

| | \–––100––––+ /––––––––––– Psychromonas ingrahamii

| | \––––71–––––+––––––––––– Vibrio fischeri

\––––68–––––+––––––––––––––––––––––––––––––––––––––––––––– Azobacteroides pseudotrichonymphae

| /––––––––––– Anaerostipes sp.

+–––––––––––––––100–––––––––––––––+––––––––––– Roseburia intestinalis

|

| /––––––––––––––––––––––– Peptoniphilus harei

| | /––––––––––– Trichomonas vaginalis G3

| /–––**100**––––+ +––––––––––– Trichomonas vaginalis Casu2

| | \––––100––––+––––––––––– Trichomonas vaginalis Moz-4

| | +––––––––––– Trichomonas vaginalis Pinna

\––––51––––+ \––––––––––– Trichomonas vaginalis Tor-A

|

| /––––––––––– Clostridium citroniae

\––––––––––86––––––––––+––––––––––– Coprococcus catus

Trichomonas – Firmicutes – Bacteria – Eukaryota – Animalia – Plantae – Archaea **designative nodes**

1. TVAG_243700.nxs /–––––––– Campylobacterales bacterium

/–––––100––––––+–––––––– Helicobacter pylori

| /–––––––– Mycobacterium colombiense

+–––––100––––––+–––––––– Saccharomonospora glauca

| /––––––––––––––– Rickettsia akari

+––82–––+ /–––––––– Escherichia coli

| \––84––+–––––––– Methylobacter tundripaludum

+––––––––––––––––––––––– Thermus thermophilus

+––––––––––––––––––––––– Dehalococcoides ethenogenes

+––––––––––––––––––––––– Geobacter bemidjiensis

+––––––––––––––––––––––– Marinitoga piezophila

+––––––––––––––––––––––– Aminomonas paucivorans

+––––––––––––––––––––––– Caldithrix abyssi

|

| /–––––––– Trichomonas vaginalis G3 (TVLF)

| +–––––––– Trichomonas vaginalis Casu2 (TVLF)

/––52––+ /––95––+–––––––– Trichomonas vaginalis Moz4 (TVLF)

| | | +–––––––– Trichomonas vaginalis Pinna (TVLF)

| +––**100**––+ \–––––––– Trichomonas vaginalis TorA (TVLF)

| | \––––––––––––––– Peptoniphilus harei

| |

| +––––––––––––––––––––––– Clostridium methylpentosum

/––––––72––––––+ | /–––––––– Peptostreptococcus anaerobius

| | +–––––100––––––+–––––––– Peptostreptococcus stomatis

/––86–––+ | | /–––––––– Bacillus methanolicus

| | | +––––––54––––––+–––––––– Brevibacillus sp.

| | | | /––––––––––––––– Haloplasma contractile

| | | +––66–––+ /–––––––– Bacteroides pectinophilus

/––58––+ | | | \––65––+–––––––– Eubacterium ventriosum

| | | | +––––––––––––––––––––––– Pinus contorta

| | | | | /–––––––– Methanocella conradii

| | | | +–––––100––––––+–––––––– Methanocella paludicola

| | | | \––––––––––––––––––––––– Apis florea

| | | \–––––––––––––––––––––––––––––– Giardia intestinalis

| | \–100––––––––––––––––––––––––––––––––––––––––– Trichomonas vaginalis G3 (II-VII, eukaryote orthologues)

| +––––––––––––––––––––––––––––––––––––––––––––––––––––– Trypanosoma brucei

| | /––––––––––––––– Pyropia yezoensis

| +–––––––––––––––––72––––––––––––––––––+ /–––––––– Ditylum brightwellii

/–––––––+ | \––99––+–––––––– Phytophthora sojae

| | +––––––––––––––––––––––––––––––––––––––––––––––––––––– Polysphondylium pallidum

| | +––––––––––––––––––––––––––––––––––––––––––––––––––––– Naumovozyma dairenensis

| | +––––––––––––––––––––––––––––––––––––––––––––––––––––– Salpingoeca sp.

| | +––––––––––––––––––––––––––––––––––––––––––––––––––––– Batrachochytrium dendrobatidis

| | +––––––––––––––––––––––––––––––––––––––––––––––––––––– Cryptococcus neoformans

| | \––––––––––––––––––––––––––––––––––––––––––––––––––––– Malassezia globosa

| \–––––––––––––––––––––––––––––––––––––––––––––––––––––––––––– Caenorhabditis elegans

\–––––––––––––––––––––––––––––––––––––––––––––––––––––––––––––––––––– Mus musculus

Trichomonas – Firmicutes – Bacteria – Eukaryota – Animalia – Plantae – Archaea **designative nodes**

1. TVAG_243710-243720.nxs

/––––––––––––––––––––––––––––––––––––––––––––––––––––––––– Clostridium symbiosum

| /––––––––––––––––––––––––––––––––––––––––––––––––– Peptoniphilus sp. oral taxon 386

| | /––––––––––––––––––––––––––––––––––––––––– Peptoniphilus lacrimalis

+––**100**––+ | /––––––––––––––––––––––––––––––––– Peptoniphilus rhinitidis

| \––**100**––+ | /––––––––––––––––––––––––– Peptoniphilus harei

| \––61–––+ | /–––––––––––––––– Trichomonas vaginalis Tor-A

/–––100––+ \––**98**–––+ +–––––––––––––––– Trichomonas vaginalis Casu2

| | \–––99–––+–––––––––––––––– Trichomonas vaginalis G3

| | | /–––––––– Trichomonas vaginalis Pinna

| | \––100––+–––––––– Trichomonas vaginalis Moz-4

| |

| | /–––––––– Treponema primitia

/–––––––+ +–––––––––––––––––––––––94–––––––––––––––––––––––+–––––––– Treponema azotonutricium

| | \––––––––––––––––––––––––––––––––––––––––––––––––––––––––– Fusobacterium sp. 3127

| \–––––––––––––––––––––––––––––––––––––––––––––––––––––––––––––––––– Lachnospiraceae bacterium

\–––––––––––––––––––––––––––––––––––––––––––––––––––––––––––––––––––––––––– Blautia hansenii

Trichomonas – Firmicutes – Bacteria – Eukaryota – Animalia – Plantae – Archaea **designative nodes**

1. TVAG_243730.nxs

/––––––––––––––––––––––––––––––––––––––––––––––––––––––––––––––––––––––––––––––––––––––––– Ashbya gossypii

|

| /–––––––––––––––––––––––––––––––––––– Peptoniphilus harei

| | /–––––––––––––––––– Trichomonas vaginalis Casu2

| /––––––**100**–––––––+ +–––––––––––––––––– Trichomonas vaginalis Tor-A

| | \–––––––91––––––––+–––––––––––––––––– Trichomonas vaginalis G3

| | +–––––––––––––––––– Trichomonas vaginalis Moz-4

| /–––––––**83**––––––––+ \–––––––––––––––––– Trichomonas vaginalis Pinna

| | | /–––––––––––––––––– Peptoniphilus indolicus

| | \––––––––––––––––70––––––––––––––––+–––––––––––––––––– Peptoniphilus sp.

\–––––––––––––––––+

|––––––––––––––––––––––––––––––––––––––––––––––––––––––––––––––––––––––– Eubacteriaceae bacterium

+––––––––––––––––––––––––––––––––––––––––––––––––––––––––––––––––––––––– Clostridium arbusti

+––––––––––––––––––––––––––––––––––––––––––––––––––––––––––––––––––––––– Acetobacterium woodii

\––––––––––––––––––––––––––––––––––––––––––––––––––––––––––––––––––––––– Acetonema longum

Trichomonas – Firmicutes – Bacteria – Eukaryota – Animalia – Plantae – Archaea **designative nodes**

1. TVAG_243740.nxs

/–––––––––––––––––––––––––––––––––––––––––––––––––––––––––––––––––––––––––––– Thalassiosira oceanica

| /––––––––––––––––––––––––––––––––––––––––––––––––––––––––––––––––– Cyanidioschyzon merolae

| | /––––––––––– Rhodomonas salina

| +–––––––––––––––––––––––––74––––––––––––––––––––––––––+––––––––––– Guillardia theta

| +––––––––––––––––––––––––––––––––––––––––––––––––––––––––––––––––– Chlamydomonas reinhardtii

| | /––––––––––– Bombus impatiens

| | /––––53––––+––––––––––– Thauera sp.

| +––––––––––––––––––––54––––––––––––––––––––+–––––––––––––––––––––– Xenopus tropicalis

| +––––––––––––––––––––––––––––––––––––––––––––––––––––––––––––––––– Micromonas sp.

| +––––––––––––––––––––––––––––––––––––––––––––––––––––––––––––––––– Ectocarpus siliculosus

| +––––––––––––––––––––––––––––––––––––––––––––––––––––––––––––––––– Bathycoccus prasinos

| | /––––––––––– Parvimonas micra

| | /––––––––––––––––––––52––––––––––––––––––––+––––––––––– Anaerococcus lactolyticus

| | | /––––––––––– Alkaliphilus metalliredigens

| | +––––––––––––––––––––56––––––––––––––––––––+––––––––––– Clostridium acidurici

| | |

| +––––67––––+ /––––––––––––––––––––––––––––––––––––––––––– Peptoniphilus duerdenii

\––––––––––+ | | /––––––––––––––––––––––––––––––––– Peptoniphilus lacrimalis

| \––––**83**––––+ | /–––––––––––––––––––––– Peptoniphilus rhinitidis

| \–––54––––+ | /––––––––––– Peptoniphilus harei

| \––––**70**––––+ +––––––––––– Trichomonas vaginalis Casu2

| | +––––––––––– Trichomonas vaginalis G3

| \––––60––––+––––––––––– Trichomonas vaginalis Moz-4

| +––––––––––– Trichomonas vaginalis Pinna

| \––––––––––– Trichomonas vaginalis Tor-A

|

| /––––––––––– Acaryochloris marina

+–––––––––––––––––––––––––57––––––––––––––––––––––––––+––––––––––– Crocosphaera watsonii

+––––––––––––––––––––––––––––––––––––––––––––––––––––––––––––––––– Desulfurobacterium thermolithotrophum

+––––––––––––––––––––––––––––––––––––––––––––––––––––––––––––––––– Acholeplasma laidlawii

+––––––––––––––––––––––––––––––––––––––––––––––––––––––––––––––––– Mesotoga prima

+––––––––––––––––––––––––––––––––––––––––––––––––––––––––––––––––– Desulfuromonas acetoxidans

+––––––––––––––––––––––––––––––––––––––––––––––––––––––––––––––––– Enhydrobacter aerosaccus

+––––––––––––––––––––––––––––––––––––––––––––––––––––––––––––––––– Caenispirillum salinarum

+––––––––––––––––––––––––––––––––––––––––––––––––––––––––––––––––– Methylomonas methanica

+––––––––––––––––––––––––––––––––––––––––––––––––––––––––––––––––– Edwardsiella tarda

\––––––––––––––––––––––––––––––––––––––––––––––––––––––––––––––––– Nitrococcus mobilis

Trichomonas – Firmicutes – Bacteria – Eukaryota – Animalia – Plantae – Archaea **designative nodes**

1. TVAG_243750.nxs

/–––––––––––––––––––––––––––––––––––––––––––––––––––––––––––––– Archaeoglobus profundus

|

| /–––––––––––––––––––––––––––––––––––––––––– Peptoniphilus harei

| | /––––––––––––––––––––– Trichomonas vaginalis Casu2

+––––––––**100**––––––––+ +––––––––––––––––––––– Trichomonas vaginalis G3

| \–––––––––78–––––––––+––––––––––––––––––––– Trichomonas vaginalis Moz-4

| +––––––––––––––––––––– Trichomonas vaginalis Pinna

| \––––––––––––––––––––– Trichomonas vaginalis Tor-A

|

/––––––––––––––––––––+ /––––––––––––––––––––– Thermoanaerobacter ethanolicus

| +–––––––––––––––––––56–––––––––––––––––––+––––––––––––––––––––– Clostridium perfringens

| +–––––––––––––––––––––––––––––––––––––––––––––––––––––––––––––– Syntrophothermus lipocalidus

| +–––––––––––––––––––––––––––––––––––––––––––––––––––––––––––––– Moorella thermoacetica

| +–––––––––––––––––––––––––––––––––––––––––––––––––––––––––––––– Deinococcus geothermalis

| | /–––––––––––––––––––––––––––––––––––––––––– Regiella sp.

| \––––––––74–––––––––+ /––––––––––––––––––––– Pseudoalteromonas undina

| \–––––––––71–––––––––+––––––––––––––––––––– Salmonella enterica

| \––––––––––––––––––––– Vibrio tubiashii

\––––––––––––––––––––––––––––––––––––––––––––––––––––––––––––––––––––––––––––––––––– Thermotoga neapolitana

Trichomonas – Firmicutes – Bacteria – Eukaryota – Animalia – Plantae – Archaea **designative nodes**

1. TVAG_243760.nxs

/––––––––––––––––––– Bacteroides thetaiotaomicron

/––––––––––––––––––99––––––––––––––––––+––––––––––––––––––– Odoribacter splanchnicus

+–––––––––––––––––––––––––––––––––––––––––––––––––––––––––– Sulfurihydrogenibium sp.

+–––––––––––––––––––––––––––––––––––––––––––––––––––––––––– Dehalococcoides sp.

+–––––––––––––––––––––––––––––––––––––––––––––––––––––––––– Syntrophus aciditrophicus

| /––––––––––––––––––– Ilyobacter polytropus

+––––––––––––––––––72––––––––––––––––––+––––––––––––––––––– Anaerococcus lactolyticus

|

| /––––––––––––––––––––––––––––––––––––––– Peptoniphilus harei

/––––––––––––––––––+ | /––––––––––––––––––– Trichomonas vaginalis Tor-A

| +–––––––**100**––––––––+ +––––––––––––––––––– Trichomonas vaginalis Moz-4

| | \––––––––100––––––––+––––––––––––––––––– Trichomonas vaginalis Casu2

| | +––––––––––––––––––– Trichomonas vaginalis G3

| | \––––––––––––––––––– Trichomonas vaginalis Pinna

| |

| +–––––––––––––––––––––––––––––––––––––––––––––––––––––––––– Thermoanaerobacter tengcongensis

| \–––––––––––––––––––––––––––––––––––––––––––––––––––––––––– Alkaliphilus oremlandii

\––––––––––––––––––––––––––––––––––––––––––––––––––––––––––––––––––––––––––––– Clostridium acidurici

Trichomonas – Firmicutes – Bacteria – Eukaryota – Animalia – Plantae – Archaea **designative nodes**

1. TVAG_243770.nxs

/–––––––––––––––––––––––––––––––––––––––––––––––––––– Coccomyxa subellipsoidea

| /––––––––––––––––––––––––––––––––––––––––––––– Anaeromyxobacter dehalogenans

| +––––––––––––––––––––––––––––––––––––––––––––– Fusobacterium sp.

| | /––––––– Roseobacter litoralis

| +–––––––––––––––––100–––––––––––––––––+––––––– Dinoroseobacter shibae

| | \––––––– Rhodobacter sp.

| +––––––––––––––––––––––––––––––––––––––––––––– Acholeplasma laidlawii

| +––––––––––––––––––––––––––––––––––––––––––––– Brachyspira murdochii

| | /––––––– Acinetobacter sp.

| | /––100––+––––––– Caenorhabditis remanei

| | | /––––––– Bombus impatiens

/––100––+ +–––––––––––––81––––––––––––––+ +––––––– Acyrthosiphon pisum

| | | \––74–––+––––––– Proteus mirabilis

| \––54––+ +––––––– Erwinia pyrifoliae

| | \––––––– Yersinia ruckeri

| +––––––––––––––––––––––––––––––––––––––––––––– Elusimicrobium minutum

| | /––––––– Hordeum vulgare

| +–––––––––––––––––78––––––––––––––––––+––––––– Dictyostelium discoideum

| | /––––––– Apis florea

| +–––––––––––––––––66––––––––––––––––––+––––––– Lactobacillus coryniformis

| +––––––––––––––––––––––––––––––––––––––––––––– Solobacterium moorei

| | /––––––– Staphylococcus massiliensis

| +–––––––––––––––––100–––––––––––––––––+––––––– Staphylococcus pseudintermedius

| +––––––––––––––––––––––––––––––––––––––––––––– Desulfotomaculum ruminis

| |

| | /––––––––––––––––––––––––––––––––––––– Peptoniphilus lacrimalis

| | | /–––––––––––––––––––––––––––––– Peptoniphilus rhinitidis

/––––––+ +––**97**–––+ | /–––––––––––––––––––––– Peptoniphilus harei

| | | \––**98**––+ | /––––––––––––––– Trichomonas vaginalis Casu2

| | | \––**87**–––+ +––––––––––––––– Trichomonas vaginalis Tor-A

| | | \––82––+ /––––––– Trichomonas vaginalis G3

| | | \––64–––+––––––– Trichomonas vaginalis Moz-4

| | |

| | +––––––––––––––––––––––––––––––––––––––––––––– Cryptobacterium curtum

| | +––––––––––––––––––––––––––––––––––––––––––––– Plesiocystis pacifica

| | | /–––––––––––––––––––––– Brachybacterium paraconglomeratum

| | +––––––––––75––––––––––+ /––––––––––––––– Intrasporangium calvum

| | | \––55––+ /––––––– Arthrobacter globiformis

| | | \––63–––+––––––– Cecembia lonarensis

| | | /––––––– Chloroflexus aurantiacus

| | \–––––––––––––––––100–––––––––––––––––+––––––– Oscillochloris trichoides

| +–––––––––––––––––––––––––––––––––––––––––––––––––––––––––––– Glycine max

| \–––––––––––––––––––––––––––––––––––––––––––––––––––––––––––– Populus trichocarpa

\––––––––––––––––––––––––––––––––––––––––––––––––––––––––––––––––––– Sorghum bicolor

Trichomonas – Firmicutes – Bacteria – Eukaryota – Animalia – Plantae – Archaea **designative nodes**

1. TVAG_243780.nxs

/––––––––– Methanocaldococcus

/––54–––+––––––––– Pyrolobus

/–––––––––––100–––––––––––+––––––––––––––––– Sulfolobus

| /––––––––– Carboxydibrachium

/––51–––+ /––––––––––100–––––––––––+––––––––– Thermoanaerobacter

| | | /––––––––– Alkaliphilus

| \–––52–––+ /–––––––89–––––––+––––––––– Clostridium

| | |

| | | /––––––––––––––––– Peptoniphilus

| \––59–––+ | /––––––––– Trichomonas vaginalis G3

| \––**100**–––+ +––––––––– Trichomonas vaginalis Casu2

/–––57–––+ \––100––+––––––––– Trichomonas vaginalis Moz4

| | +––––––––– Trichomonas vaginalis Pinna

| | \––––––––– Trichomonas vaginalis TorA

| |

| | /––––––––– Guillardia

| | /––100––+––––––––– Porphyra

/––65–––+ | /–––93–––+––––––––––––––––– Volvox

| | \–––––––––––72–––––––––––+ /––––––––– Anaerolinea

| | \–––––––93–––––––+––––––––– Thermobaculum

/––100–––+ | /––––––––– Crassostrea

| | \––––––––––––––––––––––––62––––––––––––––––––––––––+––––––––– Chloroherpeton

| | /––––––––– Desulfonatronospira

/––100––+ \––––––––––––––––––––––––––––52––––––––––––––––––––––––––––+––––––––– Pelobacter

| | /––––––––– Bombus

/––––––––+ \––––––––––––––––––––––––––––––––78–––––––––––––––––––––––––––––––––+––––––––– Haemophilus

| \––––––––––––––––––––––––––––––––––––––––––––––––––––––––––––––––––––––––––––––––––––– Xenopus

\–––––––––––––––––––––––––––––––––––––––––––––––––––––––––––––––––––––––––––––––––––––––––––––– Hydra

Trichomonas – Firmicutes – Bacteria – Eukaryota – Animalia – Plantae – Archaea **designative nodes**

1. TVAG_243790.nxs

/–––––––––––––––––––––––––––––––––––––––––––––– Peptostreptococcus anaerobius

| /–––––––––––––––––––––––––––––––––– Peptoniphilus harei

| | /––––––––––– Trichomonas vaginalis Moz-4

+––––**100**––––+ /––––58–––––+––––––––––– Trichomonas vaginalis G3

/––––**93**––––+ \––––93––––+––––––––––––––––––––––– Trichomonas vaginalis Tor-A

| +–––––––––––––––––––––––––––––––––––––––––––––– Alkaliphilus metalliredigens

| +–––––––––––––––––––––––––––––––––––––––––––––– Eubacteriaceae bacterium

/––––**78**–––––+ +–––––––––––––––––––––––––––––––––––––––––––––– Clostridium sticklandii

| | \–––––––––––––––––––––––––––––––––––––––––––––– Filifactor alocis

/––––––––––+ \––––––––––––––––––––––––––––––––––––––––––––––––––––––––– Selenomonas sp.

| |

| \––––––––––––––––––––––––––––––––––––––––––––––––––––––––––––––––––––– Sulfuricurvum kujiense

\–––––––––––––––––––––––––––––––––––––––––––––––––––––––––––––––––––––––––––––––– Ilyobacter polytropus

Trichomonas – Firmicutes – Bacteria – Eukaryota – Animalia – Plantae – Archaea **designative nodes**

1. TVAG_243800.nxs

/––––––––––––––––––––––––––––––––––––––––––––––––––––––––––––––––––––– Thermosipho africanus

+––––––––––––––––––––––––––––––––––––––––––––––––––––––––––––––––––––– Treponema vincentii

+––––––––––––––––––––––––––––––––––––––––––––––––––––––––––––––––––––– Desulfobacula toluolica

| /––––––––––––––––– Brachybacterium squillarum

+––––––––––––––––––––––––54–––––––––––––––––––––––––+––––––––––––––––– Facklamia languida

+––––––––––––––––––––––––––––––––––––––––––––––––––––––––––––––––––––– Oscillibacter valericigenes

+––––––––––––––––––––––––––––––––––––––––––––––––––––––––––––––––––––– Caldicellulosiruptor bescii

+––––––––––––––––––––––––––––––––––––––––––––––––––––––––––––––––––––– Clostridium carboxidivorans

+––––––––––––––––––––––––––––––––––––––––––––––––––––––––––––––––––––– Alkaliphilus oremlandii

|

/––––––––––––––––+ /–––––––––––––––––––––––––––––––––––––––––––––––––––– Peptoniphilus harei

| | | /––––––––––––––––– Trichomonas vaginalis Moz-4

| +––––––**100**–––––––+ /–––––––58–––––––+––––––––––––––––– Trichomonas vaginalis G3

| | | +–––––––––––––––––––––––––––––––––– Trichomonas vaginalis Pinna

| | \–––––––90––––––––+ /––––––––––––––––– Trichomonas vaginalis Tor-A

| | \–––––––69–––––––+––––––––––––––––– Trichomonas vaginalis Casu2

| |

| +––––––––––––––––––––––––––––––––––––––––––––––––––––––––––––––––––––– Dictyostelium fasciculatum

| +––––––––––––––––––––––––––––––––––––––––––––––––––––––––––––––––––––– Methanocaldococcus fervens

| \––––––––––––––––––––––––––––––––––––––––––––––––––––––––––––––––––––– Thermococcus kodakarensis

\–––––––––––––––––––––––––––––––––––––––––––––––––––––––––––––––––––––––––––––––––––––– Pyrococcus horikoshii

Trichomonas – Firmicutes – Bacteria – Eukaryota – Animalia – Plantae – Archaea **designative nodes**

1. TVAG_243810.nxs

/––––––––––––––––––––––––––––––––––––––––––––––––––––––––––––––––– Runella slithyformis

| /––––––––––– Sulfurihydrogenibium azorense

+–––––––––––––––––––––––––99––––––––––––––––––––––––––+––––––––––– Sulfurihydrogenibium sp.

+––––––––––––––––––––––––––––––––––––––––––––––––––––––––––––––––– Akkermansia muciniphila

| /–––––––––––––––––––––– Treponema sp.

+––––––––––––––––––––83––––––––––––––––––––+ /––––––––––– Legionella pneumophila

| \––––53––––+––––––––––– Melioribacter roseus

+––––––––––––––––––––––––––––––––––––––––––––––––––––––––––––––––– Thermovirga lienii

+––––––––––––––––––––––––––––––––––––––––––––––––––––––––––––––––– Rhodospirillum rubrum

+––––––––––––––––––––––––––––––––––––––––––––––––––––––––––––––––– Koribacter versatilis

+––––––––––––––––––––––––––––––––––––––––––––––––––––––––––––––––– Flexistipes sinusarabici

+––––––––––––––––––––––––––––––––––––––––––––––––––––––––––––––––– Anaerolinea thermophila

+––––––––––––––––––––––––––––––––––––––––––––––––––––––––––––––––– Streptomyces griseoaurantiacus

|

/––––––––––+ /–––––––––––––––––––––––––––––––––––––––––––––––––––––– Peptoniphilus sp.

| | | /––––––––––– Trichomonas vaginalis G3

| | | /–––100––––+––––––––––– Trichomonas vaginalis Pinna

| +––––**87**––––+ | /––––––––––– Trichomonas vaginalis Casu2

| | | /––––72––––+––––77––––+––––––––––– Trichomonas vaginalis Moz-4

| | | /–––**99**––––+ \–––––––––––––––––––––– Trichomonas vaginalis Tor-A

| | \––––**98**––––+ \––––––––––––––––––––––––––––––––– Peptoniphilus harei

| | \––––––––––––––––––––––––––––––––––––––––––– Peptoniphilus rhinitidis

| |

| +––––––––––––––––––––––––––––––––––––––––––––––––––––––––––––––––– Bacillus pseudofirmus

| +––––––––––––––––––––––––––––––––––––––––––––––––––––––––––––––––– Megasphaera sp.

| | /––––––––––– Thermoanaerobacter italicus

| \–––––––––––––––––––––––––97––––––––––––––––––––––––––+––––––––––– Thermoanaerobacterium saccharolyticum

\–––––––––––––––––––––––––––––––––––––––––––––––––––––––––––––––––––––––––––– Clostridium difficile

Trichomonas – Firmicutes – Bacteria – Eukaryota – Animalia – Plantae – Archaea **designative nodes**

1. TVAG_243820.nxs

/–––––––––––––––––––––––––––– Xenopus tropicalis

+–––––––––––––––––––––––––––– Ascaris suum

+–––––––––––––––––––––––––––– Apis florea

+–––––––––––––––––––––––––––– Mesoplasma florum

+–––––––––––––––––––––––––––– Caminibacter mediatlanticus

+–––––––––––––––––––––––––––– Hirschia baltica

+–––––––––––––––––––––––––––– Laribacter hongkongensis

/––––––––––––––––––––62––––––––––––––––––––+–––––––––––––––––––––––––––– Desulfobacter postgatei

| | /–––––––––––––– Thermosipho africanus

| +–––––54––––––+–––––––––––––– Anaerolinea thermophila

| | /–––––––––––––– Robiginitalea biformata

| +–––––60––––––+–––––––––––––– Chloroherpeton thalassium

| \–––––––––––––––––––––––––––– Conexibacter woesei

|

| /––––––––––––––––––––––––––––––––––––––––––––––––––––––––– Peptoniphilus lacrimalis

/–––––––––––––+ | /––––––––––––––––––––––––––––––––––––––––––– Peptoniphilus rhinitidis

| +–––––**99**––––––+ | /–––––––––––––––––––––––––––– Peptoniphilus harei

| | \–––––**98**––––––+ | /–––––––––––––– Trichomonas vaginalis Casu2

| | \––––––**72**––––––+ +–––––––––––––– Trichomonas vaginalis Moz4

| | \–––––50––––––+–––––––––––––– Trichomonas vaginalis Pinna

| | +–––––––––––––– Trichomonas vaginalis TorA

| | \–––––––––––––– Trichomonas vaginalis G3

| |

| +––––––––––––––––––––––––––––––––––––––––––––––––––––––––––––––––––––––– Caloramator australicus

| | /–––––––––––––– Alkaliphilus metalliredigens

| \–––––––––––––––––––––––––––79–––––––––––––––––––––––––––+–––––––––––––– Alkaliphilus oremlandii

\––––––––––––––––––––––––––––––––––––––––––––––––––––––––––––––––––––––––––––––––––––– Clostridium acidurici

Trichomonas – Firmicutes – Bacteria – Eukaryota – Animalia – Plantae – Archaea **designative nodes**

1. 243830v3.nxs

/–––––––––––––––––––––––––––– Xenopus tropicalis

+–––––––––––––––––––––––––––– Ascaris suum

+–––––––––––––––––––––––––––– Apis florea

+–––––––––––––––––––––––––––– Mesoplasma florum

+–––––––––––––––––––––––––––– Caminibacter mediatlanticus

+–––––––––––––––––––––––––––– Hirschia baltica

+–––––––––––––––––––––––––––– Laribacter hongkongensis

/––––––––––––––––––––62––––––––––––––––––––+–––––––––––––––––––––––––––– Desulfobacter postgatei

| | /–––––––––––––– Thermosipho africanus

| +–––––54––––––+–––––––––––––– Anaerolinea thermophila

| | /–––––––––––––– Robiginitalea biformata

| +–––––60––––––+–––––––––––––– Chloroherpeton thalassium

| \–––––––––––––––––––––––––––– Conexibacter woesei

|

| /––––––––––––––––––––––––––––––––––––––––––––––––––––––––– Peptoniphilus lacrimalis

/–––––––––––––+ | /––––––––––––––––––––––––––––––––––––––––––– Peptoniphilus rhinitidis

| +–––––**99**––––––+ | /–––––––––––––––––––––––––––– Peptoniphilus harei

| | \–––––**98**––––––+ | /–––––––––––––– Trichomonas vaginalis Casu2

| | \––––––**72**––––––+ +–––––––––––––– Trichomonas vaginalis Moz4

| | \–––––50––––––+–––––––––––––– Trichomonas vaginalis Pinna

| | +–––––––––––––– Trichomonas vaginalis TorA

| | \–––––––––––––– Trichomonas vaginalis G3

| |

| +––––––––––––––––––––––––––––––––––––––––––––––––––––––––––––––––––––––– Caloramator australicus

| | /–––––––––––––– Alkaliphilus metalliredigens

| \–––––––––––––––––––––––––––79–––––––––––––––––––––––––––+–––––––––––––– Alkaliphilus oremlandii

\––––––––––––––––––––––––––––––––––––––––––––––––––––––––––––––––––––––––––––––––––––– Clostridium acidurici

Trichomonas – Firmicutes – Bacteria – Eukaryota – Animalia – Plantae – Archaea **designative nodes**

1. TVAG_243830_Trichomonas.nxs

/–––––––––––––––––––––––––––––––––––––––––––––––––– Peptoniphilus harei

|

| /––––––– Trichomonas vaginalis Casu2

| /––––––––**91**––––––––––––––––––––––––+––––––– Trichomonas vaginalis Tor-A

| |

\–––––––+ /–––––––––––––––––––––––– Trichomonas vaginalis G3

| |

\–––––––**100**–––––––+ /––––––– Trichomonas vaginalis Moz-4

\–––––––**92**–––––––+––––––– Trichomonas vaginalis Pinna

Trichomonas – Firmicutes – Bacteria – Eukaryota – Animalia – Plantae – Archaea **designative nodes**

**Summary and results from phylogenetic analyses of all 27 genes of the TVLF, and analysis to elucidate relationship between *Trichomonas vaginalis* strains.**

**Matrix #taxa #char #inf.char #trees #MPT par.time boot.time**

–––––––––––––––––––––––––––––––––––––––––––––––––––––––––––––––––––––––––––––––––––––––––––––––––––––––––––––

TVAG_243570.nxs 32 872 706 2 7644 00:02:00.30 00:00:46.70

TVAG_243580.nxs 15 134 99 1 487 00:00:01.09 00:00:00.97

TVAG_243590.nxs 22 122 100 3 746 00:00:19.88 00:00:04.54

TVAG_243600.nxs 20 162 138 12 886 00:00:06.91 00:00:03.77

TVAG_243610.nxs 20 338 255 3 1544 00:00:05.51 00:00:03.38

TVAG_243620-30.nxs 11 204 122 3 365 00:00:00.23 00:00:00.22

TVAG_243640.nxs 28 448 367 1 3306 00:00:36.07 00:00:14.96

TVAG_243650.nxs 14 358 258 1 1279 00:00:01.46 00:00:12.71

TVAG_243660.nxs 18 265 213 3 1507 00:00:07.10 00:00:36.13

TVAG_243670.nxs 22 61 54 3 370 00:00:06.62 00:00:03.86

TVAG_243680.nxs 9 304 90 1 422 00:00:00.14 00:00:00.13

TVAG_243690.nxs 19 293 269 1 1857 00:00:11.63 00:00:04.21

TVAG_243700.nxs 50 312 210 1 2162 00:01:36.10 00:08:33.80

TVAG_243710-20.nxs 15 254 192 12 841 00:00:00.83 00:00:01.19

TVAG_243730.nxs 13 120 93 1 418 00:00:00.72 00:00:00.59

TVAG_243740.nxs 35 90 73 1 678 00:01:34.10 00:08:56.30

TVAG_243750.nxs 17 76 50 8 261 00:00:01.96 00:00:01.51

TVAG_243760.nxs 16 408 335 1 2024 00:00:08.46 00:00:03.16

TVAG_243770.nxs 42 121 102 6 951 00:02:10.90 00:00:44.40

TVAG_243780.nxs 26 292 205 1 1122 00:00:07.10 00:00:04.47

TVAG_243790.nxs 12 559 378 2 1968 00:00:01.96 00:00:01.61

TVAG_243800.nxs 19 127 108 1 753 00:00:06.80 00:00:03.47

TVAG_243810.nxs 26 131 107 22 797 00:00:32.33 00:00:18.60

TVAG_243820.nxs 34 215 190 2 1975 00:01:08.40 00:04:12.80

TVAG_243830.nxs 25 125 97 4 721 00:00:11.49 00:00:07.52

TVAG_Trichomonas 6 6469 41 1 612 00:00:00.04 00:00:00.08

–––––––––––––––––––––––––––––––––––––––––––––––––––––––––––––––––––––––––––––––––––––––––––––––––––––––––––––
